# Disruption of *Leishmania* flagellum attachment zone architecture causes flagellum loss

**DOI:** 10.1111/mmi.15199

**Published:** 2023-11-27

**Authors:** Clare Halliday, Laryssa Vanessa de Liz, Sue Vaughan, Jack D. Sunter

**Affiliations:** ^1^ Department of Biological and Medical Sciences Oxford Brookes University Oxford UK; ^2^ Departamento de Microbiologia, Imunologia e Parasitologia Universidade Federal de Santa Catarina Florianópolis SC Brazil

## Abstract

*Leishmania* are flagellated eukaryotic parasites that cause leishmaniasis and are closely related to the other kinetoplastid parasites such as *Trypanosoma brucei*. In all these parasites there is a cell membrane invagination at the base of the flagellum called the flagellar pocket, which is tightly associated with and sculpted by cytoskeletal structures including the flagellum attachment zone (FAZ). The FAZ is a complex interconnected structure linking the flagellum to the cell body and has critical roles in cell morphogenesis, function and pathogenicity. However, this structure varies dramatically in size and organisation between these different parasites, suggesting changes in protein localisation and function. Here, we screened the localisation and function of the *Leishmania* orthologues of *T. brucei* FAZ proteins identified in the genome‐wide protein tagging project TrypTag. We identified 27 FAZ proteins and our deletion analysis showed that deletion of two FAZ proteins in the flagellum, FAZ27 and FAZ34 resulted in a reduction in cell body size, and flagellum loss in some cells. Furthermore, after null mutant generation, we observed distinct and reproducible changes to cell shape, demonstrating the ability of the parasite to adapt to morphological perturbations resulting from gene deletion. This process of adaptation has important implications for the study of *Leishmania* mutants.

## Graphical abstract

In *Leishmania* parasites the flagellum attachment zone (FAZ) is a complex multi‐domain cytoskeletal structure that determines cell morphogenesis and pathogenicity. We identified 27 FAZ proteins and showed that deletion of two FAZ proteins resulted in a reduced cell body size, and flagellum loss in some cells.

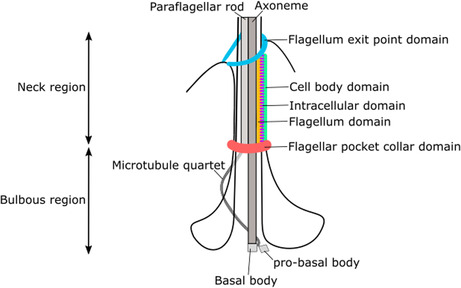

## 1 INTRODUCTION

*Leishmania* spp. are eukaryotic parasites that cause the infectious disease leishmaniasis and are transmitted between mammalian hosts via the sand fly vector (Bates, 2007). *Leishmania* spp. have a complex digenetic life cycle with developmental forms in both the mammalian host and insect vector. To adapt to the different environmental conditions of these ecological niches, *Leishmania* parasites have the ability to differentiate into different cell morphologies and cell types (Dandugudumula et al., 2022; Dostálová & Volf, 2012; Gossage et al., 2003; Sunter & Gull, 2017; Wheeler et al., 2013). There are two key cell morphologies, the promastigote form, found in the sand fly, which has an elongated ovoid cell body and a long motile flagellum, and the amastigote form, found in the mammalian host, characterised by a smaller, spherical cell body and a short immotile flagellum (Hoare & Wallace, 1966; Sunter & Gull, 2017). Despite these different morphologies, these forms share several important cellular architectural features. The overall shape of the parasite is determined by a sub‐pellicular microtubule array, and within the body are positioned single copy organelles and structures, including the nucleus, kinetoplast (concatenated mitochondrial DNA), basal body, flagellum and an invagination of the cell membrane at the base of the flagellum, the flagellar pocket (Sunter & Gull, 2017; Wheeler et al., 2011).

**FIGURE 1:**
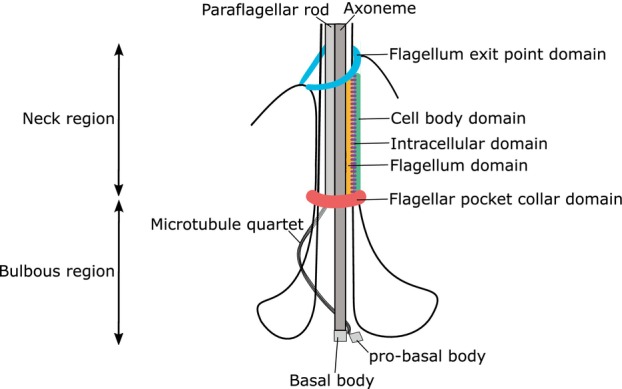
*Leishmania mexicana* flagellar pocket comprises a neck and a bulbous region. *L*. *mexicana* flagellum is laterally attached to the cell body membrane in the neck region of the flagellar pocket. The attachment is mediated through the flagellum attachment zone, comprising the flagellum exit point, cell body, intracellular, flagellum and flagellar pocket collar domains.

The sub‐pellicular microtubule array of these parasites is highly organised and runs from the anterior to the posterior of the cell. The cross‐linked, close positioning of the adjacent microtubules limits access to the cell membrane, with the site of flagellum emergence from the flagellar pocket being a critical discontinuity in the array (Ambit et al., 2011; Elias et al., 2007; Field & Carrington, 2009; Landfear & Ignatushchenko, 2001). The *Leishmania* flagellar pocket consists of a bulbous region and a neck region, and it is associated with several important cytoskeletal structures, including the microtubule quartet (MtQ), flagellum attachment zone (FAZ) and the flagellar pocket collar (Figure 1). The flagellar pocket is a critical part of the exo/endocytic system, and is an important interface between the parasite and its environment in the host and vector, in addition to being of critical for cell division and determination of cell morphology (Field & Carrington, 2009; Halliday et al., 2021).

The FAZ is a large interconnected set of fibres, filaments and junctional complexes linking the flagellum cytoskeleton via both the flagellum and cell body membranes to the specialised FAZ filament and associated MtQ (Lacomble et al., 2009; Sunter & Gull, 2016; Vickerman, 1969). The FAZ is organised into three major structural domains; (1) FAZ flagellum domain, (2) FAZ intracellular domain and (3) FAZ cell body domain (Figure 1) (Sunter & Gull, 2016). The lateral attachment of the flagellum to the cell body through the FAZ is a common feature of the kinetoplastid parasites that include *Leishmania* and *Trypanosoma brucei*. In *Leishmania*, the flagellum is only laterally attached within the flagellar pocket neck, resulting in the ‘free flagellum’ morphology, whereas in *T. brucei*, the flagellum is attached for the majority of its length (Sunter & Gull, 2016; Wheeler et al., 2016). Moreover, the architectural organisation of the *Leishmania* FAZ is more complex in comparison to the predominantly linear FAZ in trypanosomes (Corrales et al., 2021; Wheeler et al., 2016). Orthologues of FAZ proteins identified in *T. brucei* were shown to localise to five regions of the *Leishmania* flagellar pocket: (i) a ring/horseshoe at the anterior cell tip as the flagellum emerges from the cell body; (ii) a ring associated with the flagellar pocket collar; (iii) a short line within the flagellum; (iv) a short line within the cell body parallel to the flagellum; (v) an asymmetric ring surrounding the flagellar pocket neck (Corrales et al., 2021; Halliday et al., 2020; Sunter et al., 2019; Wheeler et al., 2016).

We have shown that FAZ5 in *Leishmania* is important for flagellar pocket architecture and function. In *Leishmania mexicana* promastigotes, the deletion of FAZ5 resulted in the shortening of the flagellar pocket length and loss of attachment between the flagellum and the flagellar pocket neck region (Sunter et al., 2019). These changes in the flagellar pocket were associated with a reduced rate of endocytosis. In addition, the flagellar pocket of the FAZ5 null mutant amastigote had lost the typical two‐part structure, with the flagellar pocket neck region missing, and only the constriction at the distal end of the neck remaining. The FAZ and flagellar pocket neck changes in the FAZ5 null mutant were associated with a loss of pathogenicity in the mouse and an inability to develop and proliferate in the sand fly (Sunter et al., 2019).

The deletion of FAZ2, a FAZ filament protein, was found to cause anterior cell tip morphogenesis defects in *L. mexicana* (Halliday et al., 2020). The membrane organisation at the anterior cell tip was disrupted, resulting in FAZ‐mediated flagellum to flagellum connections causing delays in cell segregation. Meanwhile, they were unable to develop and proliferate in sand flies and unable to persist infection in mice (Halliday et al., 2020). Most recently, a FAZ7 paralogue, FAZ7B, which localises at the cell body side of FAZ, was found to disrupt cell division, cell morphogenesis, flagellar pocket structure and function when deleted. The proliferation and pathogenicity of FAZ7B null mutant was also reduced as seen with other FAZ mutants (Corrales et al., 2021).

The knowledge that the *Leishmania* FAZ is much reduced in size, yet critical to cell morphology, function and pathogenicity, raises the question of whether there is a reduced complement of FAZ proteins in *Leishmania* and whether there is a variant distribution in comparison to trypanosomes. To provide an in‐depth understanding of the *Leishmania* FAZ, we screened the localisation and function of the *Leishmania* orthologues of *T. brucei* FAZ proteins identified in the genome‐wide protein tagging project TrypTag (Dean et al., 2017; Billington et al., 2023). Of the 96 TrypTag FAZ candidate proteins, we identified 27 FAZ proteins with five localisation patterns within the flagellum and flagellar pocket neck in *Leishmania*. Our deletion analysis showed that FAZ27 and FAZ34, found in the flagellum FAZ domain, were required for flagellum attachment. Intriguingly, in the weeks of growth following null mutant generation, there were distinct and reproducible changes to cell shape, demonstrating adaptation to the morphological perturbations induced by gene deletion. Such adaptations are revealed through morphological mutants, but this adaptation process will have important implications for the study of null mutants in this and related organisms.

## 2 RESULTS

### 2.1 57 orthologues of *T. brucei* FAZ proteins identified in *L. mexicana*

A cohort of 96 FAZ proteins was identified in *T. brucei* by TrypTag (Billington et al., 2023). We organised these proteins into seven categories based on their localisation patterns in *T. brucei*: (1) Full length (23 proteins), (2) Full length distal enriched (18 proteins), (3) Full length proximal enriched (3 proteins), (4) Distal only (17 proteins), (5) Proximal only (3 proteins), (6) FAZ‐ER (9 proteins), and (7) Complex (23 proteins) (Figure S1). The complex category was assigned to those with additional localisations (except cytoplasm). Protein features were collated for the 96 *T. brucei* FAZ proteins (Table S1). Of the 96 proteins, 39 had at least 1 PFAM domain. Most of PFAM domains were restricted to individual proteins; however, one PFAM domain, a TerD domain which encodes calcium binding sites, was found in both FAZ28 and FAZ30. Another domain, found in three proteins, CC2D, FAZ22 and FAZ35 (synaptogamin), was a C2 domain, which is important in calcium‐binding and membrane‐targeting processes (Davletov & Südhof, 1993; Zhou et al., 2011, 2018). 18 proteins had transmembrane domains; of these, 9 were FAZ‐ER proteins, including VAMP‐associated protein (TbVAP), which was found to be important for FAZ and flagellar pocket ER domain maintenance (Lacomble et al., 2012). OrthoMCL was used to identify which of the *T. brucei* FAZ proteins were present in *L. mexicana* (Fischer et al., 2011). Out of the 96 FAZ proteins identified in *T. brucei*, 61 had orthologues in *L. mexicana*; however, due to apparent FAZ gene duplication in *T. brucei*, there were only 57 unique *L. mexicana* orthologues (Table S1).

### 2.2 Localisation screen identified 27 FAZ proteins in *L. mexicana*

To aid identification of FAZ proteins in *L. mexicana*, we generated two marker cell lines in which either FLABP or FAZ2 were endogenously tagged with mCherry, defining the flagellum FAZ domain and the cell body FAZ domain respectively (Wheeler et al., 2016). 54 cell lines were successfully generated by endogenous tagging of the different FAZ protein candidates in *L. mexicana* with an mNeonGreen fluorescent protein at the N or C‐terminus in either the FLABP::mCherry or mCherry::FAZ2 marker cell line. We were unable to generate cell lines for three of the potential FAZ proteins FAZ22 (LmxM.36.4330), FAZ20 (LmxM.28.1650), and LmxM.21.0940. A total of 25 out of the potential 54 FAZ proteins examined were found to localise to the FAZ in *L. mexicana*, as determined by their position relative to the known FAZ protein markers (Table S2). This gives a total of 27 FAZ proteins when the FAZ protein markers were included. The 27 FAZ proteins had five distinct localisation patterns in G1 cells: Class 1) Short line co‐localising with FLABP, Class 2) Short line co‐localising with FAZ2, Class 3) A ring/horseshoe distal to the pocket collar region, Class 4) A ring‐like shape associated with the flagellum exit point, Class 5) Short line parallel to the flagellum with a ring/horseshoe. In addition, a set of proteins which localised to the FAZ but were also found elsewhere in the cell, for instance, at the basal body or cell tip were classified as proteins with a complex localisation.

**FIGURE 2:**
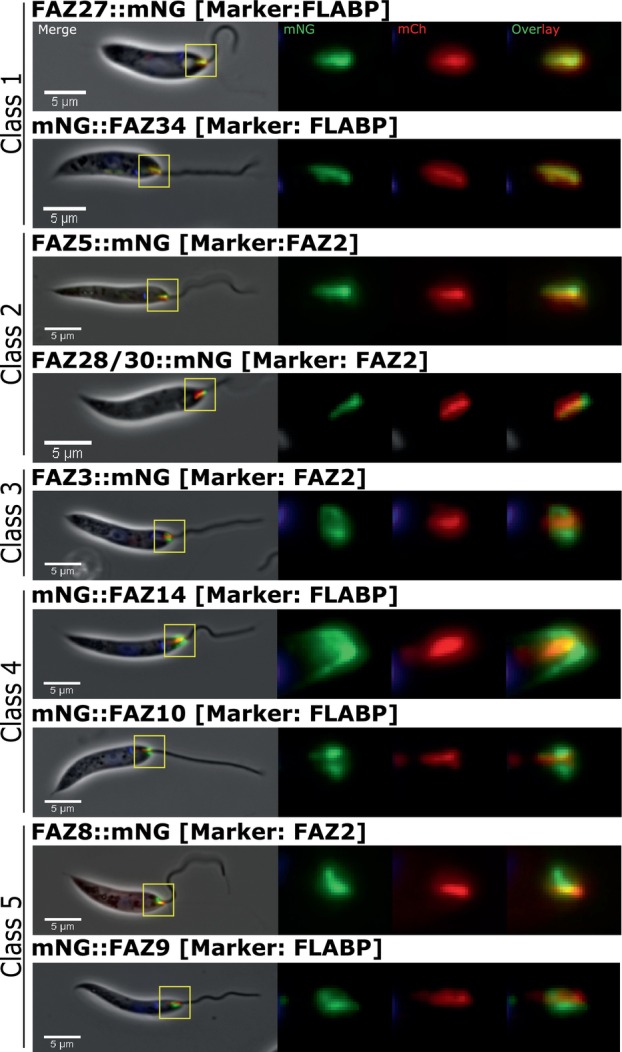
FAZ proteins in *Leishmania mexicana* were classified into five classes based on their localisation patterns. *L*. *mexicana* FAZ proteins were endogenously tagged with mNeonGreen and classified based on their locations. Known FAZ proteins, FAZ2 and FLABP were endogenously tagged with mCherry to act as markers of the FAZ. FAZ2 localised to a short linear structure on the cell body side, and FLABP localised to a linear structure within the flagellum. Class 1 proteins (FAZ27 and FAZ34) co‐localised with FLABP. Class 2 proteins (FAZ5 and FAZ28/30) co‐localised with FAZ2. The only Class 3 protein FAZ3 localised to a ring/horseshoe at the flagellar pocket collar region. Class 4 proteins (FAZ14 and FAZ10) localised to a ring/horseshoe at the flagellum exit point. Class 5 proteins (FAZ8 and FAZ9) localised to the cell body FAZ domain and collar region. From left, an overlay of the phase contrast (grey), mNG tagged protein (green), mCh tagged FAZ marker (red) and Hoechst DNA (blue) then mNG only, and mCherry only, the far right is an overlay of mNG and mCherry. Scale 5 μm. Other FAZ proteins are shown in Figure S2.

Class 1) FLAM3, FAZ27, FAZ32, and FAZ34 localised to a short line parallel to the flagellum that co‐localised with FLABP::mCherry (Figures 2 and S2) (Subota et al., 2014; Sun et al., 2013; Wheeler et al., 2016). ClpGM6 was expressed in the mCherry::FAZ2 marker cell line and localised to a short line parallel to the flagellum adjacent to the FAZ2 marker (Figure S2). The localisation pattern of these proteins is consistent with the flagellum FAZ domain.

Class 2) Three proteins, CC2D, FAZ28/30, and FAZ5 localised to a short line parallel with the flagellum and co‐localised with mCherry::FAZ2, with the FAZ28/30 signal extending beyond the distal end of the mCherry::FAZ2 signal (Figures 2 and S2). The localisation pattern of these proteins is consistent with the cell body FAZ domain.

Class 3) Only one protein, FAZ3, localised to a ring/horseshoe around the flagellar pocket collar region. This localisation was clearly distinct from mCherry::FAZ2 (Figure 2).

Class 4) Six proteins FAZ14, FAZ10, Kinesin X2D, FAZ6, FAZ40, and FAZ12, localised to a ring/horseshoe at the flagellum exit point (Figures 2 and S2). In these cell lines, the tagged protein localised on both sides of the distal end of the FLABP::mCherry signal and there was variation in the specific localisation pattern. FAZ14 and FAZ40 had a dome‐like appearance, which extended further into the cell body from the flagellum exit point (Figures 2 and S2). Whilst FAZ10 had a ring‐like appearance, as expected (Wheeler et al., 2016) (Figure 2). FAZ12, FAZ6 and Kinesin X2D had a similar horseshoe/ring‐like appearance around the exit point (Figure S2).

Class 5) Four proteins, FAZ8, FAZ1, FAZ29 and FAZ9, localised to a short line parallel with the flagellum and a ring/horseshoe (Figures 2 and S2). This localisation pattern resembled an ‘L' shape with a longer signal running parallel to the flagellum co‐localising with mCherry::FAZ2 marker as seen for FAZ8 and FAZ29 or appearing adjacent to FLABP::mCherry as seen for FAZ1 and FAZ9 (Figures 2 and S2).

Six proteins, KMP11, LmxM.32.1035, FAZ24, Kinesin 13–5, LmxM.33.0190 and FAZ36 had a FAZ signal and an additional localisation and classified as complex (Figure S3). KMP11 co‐localised with FLABP::mCherry, suggesting it localises to the flagellum FAZ domain, in addition to a signal from the flagellar cytoplasm, the posterior cell tip and the kinetoplast. LmxM.32.1035 co‐localised with FLABP::mCherry but with a much shorter signal length. It was also present in the anterior cytoskeleton. FAZ24 localised adjacent to FLABP::mCherry, suggesting FAZ24 is found in the cell body FAZ domain along with signals in the basal body and the posterior cell tip. Kinesin 13–5 had a broad signal that co‐localised with the FLABP::mCherry marker, with one edge extending beyond FLABP. A Kinesin 13–5 signal was also asymmetrically positioned to one side of the flagellar pocket, with additional cytoplasmic and basal body signals. LmxM.33.0190 co‐localised with FLABP::mCherry, with additional cytoplasm and flagellar cytoplasm signals. FAZ36 localised adjacent to FLABP::mCherry with additional basal body, cytoplasm and flagellar cytoplasm signals.

Finally, 27 candidate proteins were found not to localise to the FAZ but instead localised to a variety of different cellular locations, including the endoplasmic reticulum, cytoskeleton, basal body, endosome/lysosome and cytoplasm (Table S3; Figure S4). Those proteins with an endoplasmic reticulum signal in *Leishmania* also are localised to the endoplasmic reticulum in *T. brucei*, which is associated with the FAZ (Billington et al., 2023).

### 2.3 Screen of FAZ null mutants identified six FAZ proteins important for cell morphogenesis

27 FAZ proteins in *L. mexicana* were identified in the localisation screen, and for our functional analysis, we focused on the 21 proteins with FAZ‐only localisations. We generated null mutants for 20 of the 21 FAZ protein targets set in the cell line expressing FLABP::mCherry, except for FLABP, which was deleted in the mCherry::FAZ2 marker cell line. In all cases, gene deletion was confirmed by PCR (Figure S5a–u). We were unable to successfully generate a null mutant for ClpGM6 (LmxM.27.0490), suggesting an intrinsically essential function. The FAZ has an important role in cell morphogenesis and cell cycle progression (Corrales et al., 2021; Halliday et al., 2020; Sunter et al., 2019). To assess whether the deletion of individuals within our FAZ protein cohort affected these processes, we (i) quantified atypical cell types and cell cycle progression and (ii) evaluated changes to morphology by measuring flagellum and cell body lengths in 1F1K1N (G1) cells.

**FIGURE 3:**
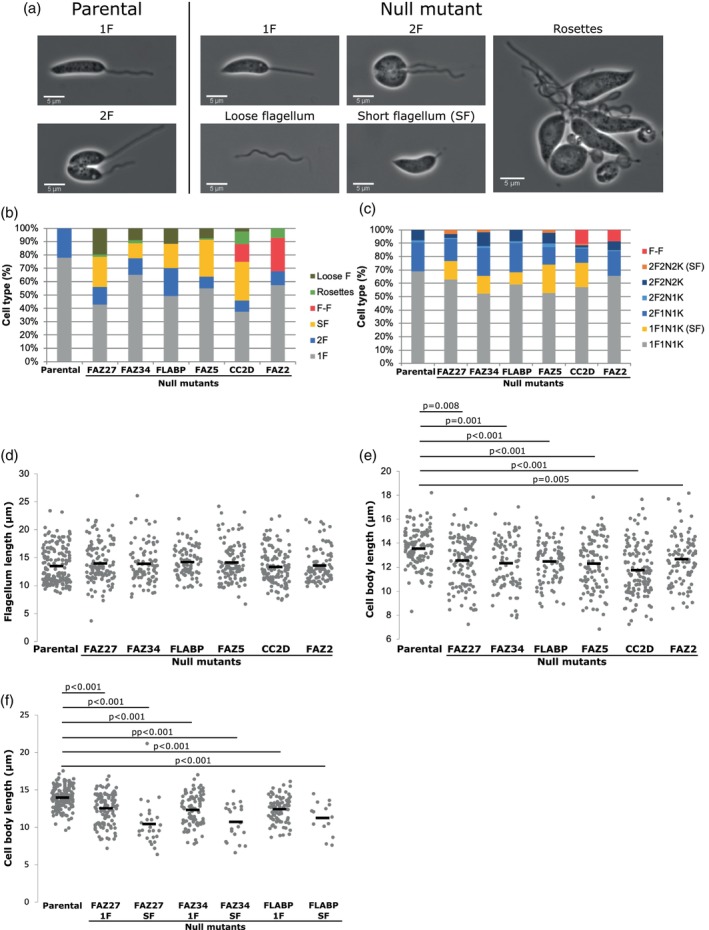
Deletion of FAZ27, FAZ34, FLABP, FAZ5 and CC2D resulted in loose flagella and cells with short flagella. (a) Example images of cells with one flagellum (1F), two flagella (2F), short flagellum (SF), loose flagellum and rosettes observed in culture. (b) Quantitation of cell types of parental cells and the null mutants of FAZ27, FAZ34, FLABP, FAZ5, CC2D and FAZ2. Percentages were calculated from number of cell types seen direct from culture ≥100 cells. Loose F: loose flagellum. F‐F: flagellum‐flagellum connection. SF: short flagellum. 2F: two flagella. 1F: one flagellum. (c) Quantitation of cell cycle positions seen in parental and the null mutants of FAZ27, FAZ34, FLABP, FAZ5, CC2D and FAZ2. Cell cycle stages include 1F1N1K, 2F1N1K, 2F2N1K, 2F2N2K and 1F1N1K and 2F2N2K cells with short flagella (SF) ‐ ≥151 cells were counted (d,e) Length of flagellum (d) and cell body (e) of parental, FAZ27, FAZ34, FLABP, FAZ5, CC2D and FAZ2 null mutant cell lines. Each dot represents the length measurement for an individual cell, and the mean (black bar and number above each cell line) was calculated from these length measurements (≥86 cells). (f–h) Mean cell body length of parental and FAZ27 (f), FAZ34 (g) and FLABP (h) null mutants with one flagellum (1F) and short flagellum (SF). *p*‐values were calculated using Kruskal–Wallis test followed by Dwass–Steel–Critchlow–Fligner Test.

- Upon initial observations of the null mutants by light microscopy, we noticed that the FAZ27, FAZ34, FLABP, FAZ2, FAZ5 and CC2D null mutants had atypical cell morphologies, including short flagellum cells (SF), loose flagella, flagellum to flagellum connections (F‐F) and cell rosettes. We quantified the presence of these different cell types directly from culture (Figure 3a,b). We observed fewer 1F and 2F cells for these null mutants and more atypical cell types, including cell rosettes, flagellum‐to‐flagellum connections and short flagellum cells.

Next, to determine whether there were changes in the cell cycle, the cell cycle progression was analysed for all null mutants generated based on the flagellum, kinetoplast and nucleus configuration (Figures 3c and S6a). At the start of the cell cycle, a G1 *Leishmania* cell has one flagellum, one kinetoplast and one nucleus—1F1N1K. The first indication of cell cycle progression is the appearance of a second flagellum followed by nucleus and kinetoplast duplication and segregation, with a post‐mitotic cell having a 2F2N2K configuration. For the vast majority of null mutants, the numbers of the different cell cycle stages present were similar to that of the parental cells, with only minimal changes (Figure S6a).

For the FAZ27, FAZ34, FLABP, CC2D and FAZ5 null mutants, additional cell types were observed consistent with our observations from culture (Figure 3c). 1F1N1K and 2F2N2K cells with short flagella were seen for the FAZ27, FAZ34, CC2D and FAZ5 null mutants; however, for the FLABP null mutant, short flagellum cells were not observed among those with a post‐mitotic 2F2N2K configuration. If the short flagellum cell categories were considered as either 1F1N1K or 2F2N2K cells, the numbers of the different cell cycle stages present for the FAZ27, FAZ34, FLABP and FAZ5 null mutants were similar to the parental cells (Figure 3). For the FAZ2 null mutant, ~9% of cells had a flagellum‐to‐flagellum connection as previously described (Halliday et al., 2020), and if these cells were considered as two incompletely separated 1F1K1N cells, the numbers of the different cell cycle stages present for the FAZ2 null mutant was similar to the parental cells. While for the CC2D null mutant, flagellum‐to‐flagellum cells were also seen alongside short flagellum cells (Figure 3). In this case, if the flagellum‐to‐flagellum cells were considered as two incompletely separated 1F1K1N cells and the short flagellum cell categories were considered as either 1F1N1K or 2F2N2K cells, then the majority of the CC2D null mutant cells had a 1F1K1N configuration, with a drop in the other cell cycle stages, suggesting a delay in the early stages of cell cycle progression.

ii To assess morphological changes, flagellum and cell body lengths were measured for 1F1N1K cells excluding those with a short flagellum. In the parental cells, flagellum length ranged from 8 to 23 μm with a mean of 13.5 μm, and all the null mutants had a similar mean flagellum length (Figures 3d and S6b,c). In the parental cells, the 1F1N1K cell body lengths ranged from 8 to 18 μm long with a mean of 13.6 μm and for the majority of null mutants, the mean cell body length was similar (Figures 3e and S6d,e). However, for the FAZ27, FAZ34, FLABP, FAZ5, CC2D and Kinesin X2D null mutants, the mean length of 1F1N1K cells was shorter by 1–1.5 μm. As many of these null mutants also had short flagellum cells, we examined the cell length of short flagellum cells for the FAZ27, FAZ34 and FLABP null mutants to assess if the cell length is also affected in this cell type. For these null mutants, the short flagellum cells were even shorter (Figure 3f). Overall, this shows that the cell morphology is altered in the FAZ27, FAZ34, FLABP, FAZ5 and CC2D null mutants.

### 2.4 Deletion of FAZ proteins caused defects in FAZ assembly

**FIGURE 4:**
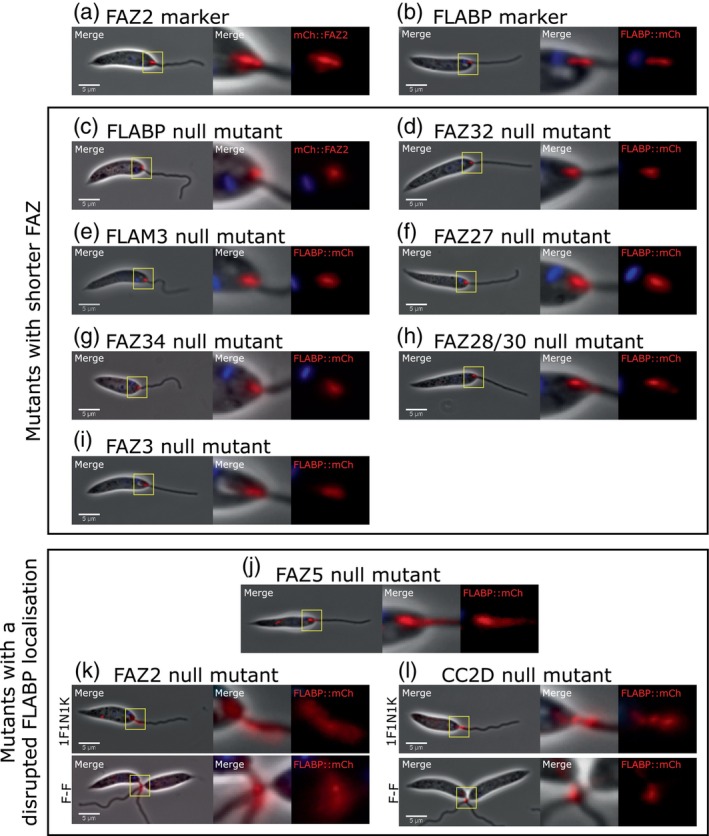
Deletion of specific FAZ proteins disrupted FAZ assembly. (a) Parental marker cell line expressing mCh::FAZ2; (b) Parental marker cell line expressing FLABP::mCh; (c–i) Deletion of FAZ proteins with a shorter FLABP signal. (c) FLABP null mutant expressing mCh::FAZ2; (d) FAZ32 null mutant expressing FLABP::mCh; (e) FLAM3 null mutant expressing FLABP::mCh; (f) FAZ27 null mutant expressing FLABP::mCh; (g) FAZ34 null mutant expressing FLABP::mCh; (h) FAZ28/30 null mutant expressing FLABP::mCh; (i) FAZ3 null mutant expressing FLABP::mCh; (j–l) Deletion of FAZ proteins that disrupted FLABP localisation. (j) FAZ5 null mutant expressing FLABP::mCh; (k) FAZ2 null mutant expressing FLABP::mCh, including flagellum‐to‐flagellum (F‐F) cells; (l) CC2D null mutant expressing FLABP::mCh, including F‐F cells. Example images of cells (Merge) contain phase (grey), mCherry (red) and Hoechst 33342 (blue). Enlarged FAZ region on the right shows merge and mCherry (red) only. Scale 5 μm.

To gain an insight into the role of these FAZ proteins in FAZ assembly and maintenance, we assessed changes in the localisation of the FAZ marker proteins (mCherry::FAZ2 or FLABP::mCherry) between the parental cells and the null mutants. In the parental cells, FLABP::mCherry and mCherry::FAZ2 localised as a short line in the flagellum or cell body respectively (Figure 4a,c). For 10 of the null mutants, we observed no difference in FLABP::mCherry localisation (Figure S7a–j); however, for the other null mutants, we saw either a reduction in the length or a disrupted localisation of the FAZ protein marker. For the FLAM3, FAZ32, FAZ27, FAZ34, FAZ28/30 and FAZ3 null mutants, the FLABP::mCherry signal was noticeably shorter (Figure 4d‐i), and for the FLABP null mutant, the mCherry::FAZ2 signal was also shorter (Figure 4b).

For the FAZ5, CC2D and FAZ2 null mutants, the FLABP::mCherry protein was mislocalised to the region of the flagellum beyond the anterior cell tip (Figure 4j‐l). For the FAZ5 null mutant, the FLABP localisation pattern was similar to that seen in the previous FAZ5 study (Sunter et al., 2019). Moreover, for CC2D and FAZ2 null mutants, the FLABP::mCherry signal was also seen at the flagellum‐to‐flagellum connection point, with the FLABP::mCherry localisation pattern in the FAZ2 null mutant similar to the previously published description (Figure 4k,l) (Halliday et al., 2020).

### 2.5 FAZ27 and FAZ34 null mutant phenotype changed over time

**FIGURE 5:**
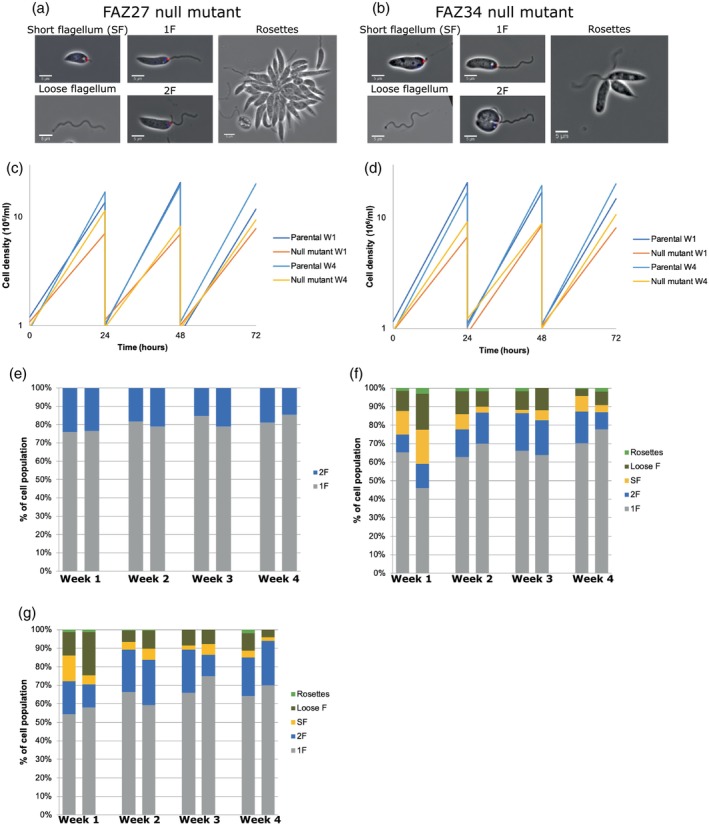
Phenotype of FAZ27 and FAZ34 null mutants changed over time. (a,b) Re‐generated FAZ27 and FAZ34 null mutants had the same mutant phenotypes seen previously ‐ loose flagella, short flagellum cells and rosettes. Cells expressed FLABP::mCh marker. (c,d) Growth curves for FAZ27 null mutant (c) and FAZ34 null mutant (d) measured in week 1 and 4. (e) Percentage of different cell types seen in culture for parental cells. (f) Percentage of cell types seen in culture for FAZ27 null mutant. (g) Percentage of cell types seen in culture for FAZ34 null mutant. For each experiment ≥100 cells were counted in week 1, 2, 3 and 4. Loose F: loose flagellum. SF: short flagellum. 2F: two flagella. 1F: one flagellum.

Previous studies of the *Leishmania* FAZ have focused exclusively on FAZ proteins found in the cell body FAZ domain (Halliday et al., 2020; Sunter et al., 2019); therefore, we decided to determine the function of FAZ proteins localised to the flagellum FAZ domain. Our screen indicated that the deletion of FAZ27 and FAZ34 had a cell morphology and FAZ assembly phenotype. To ensure the phenotypes previously seen in the deletion screen were reproducible, FAZ27 and FAZ34 null mutant cell lines were re‐generated in the parental cell line. The FAZ27 and FAZ34 null mutant cells had the same phenotype as previously observed, with loose flagella and cells with a short flagellum (Figure 5a,b).

During the observations of FAZ27 and FAZ34 null mutants, we noticed that the mutant phenotype appeared to change over time (Figure 5c‐g). We analysed the growth rate of the cells in weeks 1 and 4 after recovery from transfection (Figure 5c,d). The parental doubling time was consistent between weeks 1 and 4 (6.1 h vs. 5.7 h); however, for both the FAZ27 and FAZ34 null mutant, there was a slight decrease in doubling time between weeks 1 and 4 (FAZ27–8.7 h ± 0.42 vs. 7.2 h ± 0.41, *p* = 0.024; FAZ34–8.1 h ± 0.46 vs. 7.0 h ± 0.56, *p* = 0.45).

To understand the phenotype changes in more detail, the re‐generated FAZ27 and FAZ34 null mutants alongside the parental cell line were imaged at the same time point, in duplicates, every week over a 4‐week period (Figure 5c–g). We analysed the changes in the different cell types seen over this 4‐week period. For the parental cells, we observed a slight increase in the number of 1F cells and a concomitant decrease in 2F cells (Figure 5e). In the FAZ27 null mutant cell line, the percentage of loose flagella reduced from week 1 to week 4 (Figure 5f). Cells with a short flagellum were seen less often as time progressed, reducing in proportion from week 1 to week 2 and remaining relatively stable into week 4 (Figure 5f). In comparison, 1F and 2F cells increased over time (Figure 5f). FAZ34 null mutants behaved similarly, with a decrease in loose flagella and short flagellum cell numbers matched by an increase in 1F and 2F cells over the time (Figure 5g). This shows that there is a change in the mutant phenotype over time in culture.

**FIGURE 6:**
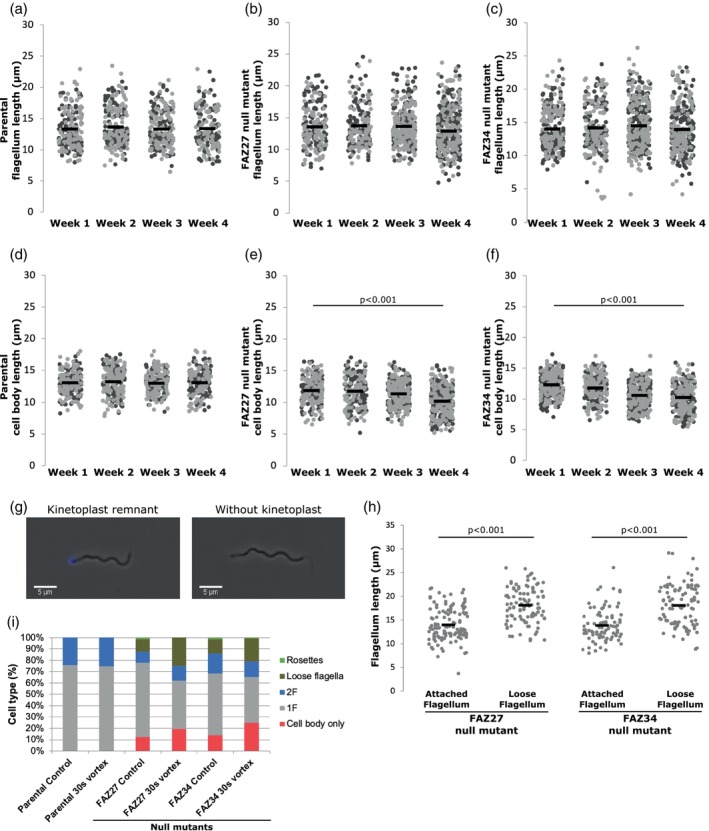
Cell body of FAZ27 and FAZ34 null mutants shortened over time in culture. A‐C) Flagellum length in parental (a), FAZ27 null mutant (b) and FAZ34 null mutant (c). (d–f) Cell body length in parental (d), FAZ27 null mutant (e) and FAZ34 null mutant (f). Each dot represents the length measurement of an individual cell, and the mean (black bar and numbers above each cell line) was calculated from these length measurements. (g) Example images of flagellum with kinetoplast remnant and flagellum without kinetoplast. Overlay of the phase contrast (grey) and Hoechst 33342 (blue). Scale 5 μm. (h) Length of attached and loose flagella from FAZ27 and FAZ34 null mutants. Each dot represents the length measurement of an individual flagellum, and the mean (black bar) was calculated from these length measurements. ≥25 cells were counted. *p*‐values were calculated using Kruskal–Wallis test followed by Dwass–Steel–Critchlow–Fligner Test. (i) Percentage of parental, FAZ27 and FAZ34 null mutant cell types measured at rest and after 30s vortex. 2F: two flagella. 1F: one flagellum. The different shades of grey indicate the replicates (*N* = 2). *p*‐values were calculated using the Kruskal–Wallis test followed by Dwass–Steel–Critchlow–Fligner Test (for Figure 6a‐f) and independent samples t‐test or Mann–Whitney test (Figure 6h).

To assess the morphology changes in detail, we measured flagellum and cell body lengths of 1F1N1K cells (Figure 6), which were captured at the same time point in duplicates weekly over the same 4‐week period. The mean of both sets of lengths were calculated (excluding short flagellum cells) from each week. The mean flagellum length of the parental cells ranged from 13.2 to 13.7 μm, with the mean cell body lengths ranging from 12.9 to 13.3 μm (Figure 6a,d). The FAZ27 null mutant mean flagellum lengths ranged from 12.9 to 14.1 μm over 4 weeks with no obvious pattern (Figure 6b). However, the cell body length gradually reduced in mean length by ~2 μm from week 1 to week 4 (Figure 6e). For the FAZ34 null mutant, the flagellum length also showed no large changes, with the mean varying from 13.7 to 14.5 μm (Figure 6c). Like the FAZ27 null mutant, the mean cell body length shortened from week 1 to week 4 by ~2 μm (Figure 6f). The cell body length reduction occurred during the same time window as the reduction in the number of loose flagella and cells with a short flagellum and an increase in growth rate, suggesting that these are adaptations linked to faster growth.

To confirm that the mutant phenotype observed was the consequence of FAZ27 and FAZ34 deletion, add‐back cell lines were generated. The add‐back cell lines expressed FAZ27 and FAZ34 tagged at the N‐terminus with mNeonGreen fluorescent protein, which localised to the FAZ as expected (Figure S8a). To check that the add‐back cell lines restored the parental phenotype, the parental, FAZ null mutants and add‐back cell lines were compared to the null mutants with a less severe phenotype after 4 weeks of growth.

For the FAZ27 add‐back, cells with a short flagellum and loose flagella were not observed, and the percentages of 1F and 2F cells were similar to the parental (Figure S8b). For the FAZ34 add‐back, cells with a short flagellum and loose flagella were also eliminated, with the proportions of cell types observed very similar to the parental cell line (Figure S8c). This demonstrated that the add‐back of the deleted protein restored the parental phenotype, indicating that the phenotype observed was the consequence of FAZ27 and FAZ34 loss.

### 2.6 Mechanical stress contributed to flagellum loss

To assess whether there was a relationship between flagellum length and flagellum loss, the length of the flagellum in 1F cells and loose flagella was measured for cells in week 1 after recovery from transfection. For both the FAZ27 and FAZ34 null mutant, the mean loose flagellum length was longer than the attached flagellum length (Figure 6h); however, this may be due to the lack of recovery of short loose flagella after centrifugation. Moreover, we found that many flagella had a kinetoplast attached, as indicated by a Hoechst 33342 stained structure at one end (Figure 6g), with ~29% (*N* = 30) and ~26% (*N* = 25) of loose flagella having an attached kinetoplast in the FAZ27 and FAZ34 null mutants respectively. This suggests that in these mutants, the entire flagellum, including the basal body is detached from the cells.

A potential explanation for the loose flagella and cells with a short flagellum in the FAZ27 and FAZ34 null mutants was that there was a weakened flagellum attachment which was more readily broken due to the mechanical stress of the flagellum beating, releasing the flagellum. To determine if the flagellum attachment was weakened and susceptible to mechanical stress, the mutants were subjected to a defined period of vortexing and the different cell types before and after were counted. The null mutants were assessed for ‘flagella loss’ in week 1 since they had a greater number of loose flagella, potentially indicating a greater susceptibility to flagella loss at this time. For the parental cells, the proportion of cell types did not change before and after vortexing (Figure 6i). For both the FAZ27 and FAZ34 null mutant, the proportion of 1F cells reduced substantially after vortexing, with a concordant increase in short flagellum cells and loose flagella (Figure 6i). The increase in short flagellum cells and loose flagella demonstrated that mechanical stress can contribute to flagellum loss in the null mutant cells.

### 2.7 Deletion of FAZ27 and FAZ34 disrupted FAZ and flagellar pocket organisation

FAZ27 and FAZ34 deletion resulted in flagellum loss in a proportion of cells, which indicates that the eponymous function of the FAZ to maintain flagellum attachment was impacted. To understand the causes of this loss of flagellum attachment, the molecular structure of the FAZ was investigated. The following FAZ proteins were chosen to represent the different FAZ domains: FLAM3 for the flagellum domain, FLABP and FAZ5 for the flagellum and cell body side of the membrane domain, respectively, FAZ2 for the cell body domain, FAZ3 for the collar domain and FAZ10 for the exit domain (Figures 2 and S2).

**FIGURE 7:**
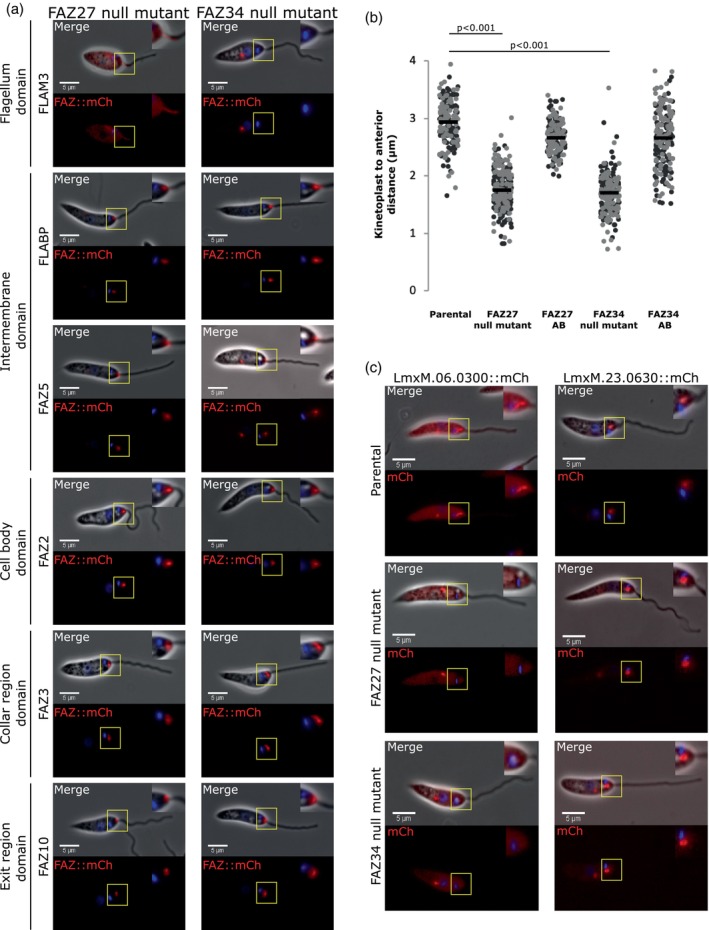
Deletion of FAZ27 and FAZ34 disrupted FAZ assembly and flagellar pocket shape and organisation. (a) FLAM3, FLABP, FAZ5, FAZ2, FAZ3 and FAZ10 were tagged with mCherry individually in the FAZ27 and FAZ34 null mutants. Top – merge containing phase (grey), mCherry (red) and Hoechst 33342 (blue); Bottom – mCherry (red) and Hoechst 33342 (blue) only. Inserts included shows an enlarged image of FAZ region. Scale 5 μm. (b) Kinetoplast to anterior cell tip distances for FAZ27 and FAZ34 null mutants compared to the parental and add‐back cell lines. Each dot represents the length measurement of an individual cell, and the mean (black bar) was calculated from these length measurements (≥100 1F1N1K cells). The different shades of grey indicate the replicates (*N* = 2). *p*‐values were calculated using Kruskal–Wallis test followed by Dwass–Steel–Critchlow–Fligner Test. (c) Widefield images showing flagellar pocket markers tagged with mCherry in parental and null mutants. Top – overlay of phase (grey), Hoechst (Blue) and mCherry (red) combined; Bottom – overlay of Hoechst 33342 (blue) and mCherry (red) only. Scale 5 μm.

For the FAZ27 null mutant, FLAM3 was mis‐localised to the cytoplasm and the proximal region of the flagellum and did not localise to a short linear structure in the flagellum, as seen in the parental cells (Figure 7a). FLABP and FAZ5, the membrane proteins, changed from a linear to a short stub‐like signal, which appeared at the distal end of the neck close to the flagellum exit point (Figure 7a). FAZ2 was also stub‐like at the anterior tip of the cell body but localised slightly away from the anterior cell tip (Figure 7a). Meanwhile, FAZ3 and FAZ10 signals showed no change with their ring/horseshoe signals at the collar region and exit point (Figure 7a).

For the FAZ34 null mutant, all but one FAZ protein followed the same pattern as the FAZ27 null mutant. The position of the intracellular domain (FLABP and FAZ5), cell body domain (FAZ2), collar domain (FAZ3) and exit domain (FAZ10) FAZ proteins appeared identical to that of FAZ27 null mutant (Figure 7a). However, the flagellum domain protein FLAM3 signal was not seen within or close to the FAZ region and instead localised to spots within the cell body possibly associated with the endocytic system (Figure 7a).

The changes to the FAZ in FAZ27 and FAZ34 null mutants could cause a reduction in the size of the attachment interface between the flagellum and the flagellar pocket neck, leading to flagellum loss. Previous studies in FAZ5 and FAZ2 deletion cell lines showed that the flagellar pocket size was reduced (Halliday et al., 2020; Sunter et al., 2019). To assess if FAZ27 and FAZ34 loss had a similar effect, the distance between the kinetoplast and the anterior cell tip was measured. In both the null mutants, the kinetoplast was positioned closer to the anterior cell tip than in comparison to the parental and add‐back cell lines (Figure 7b). This suggests that the loss of FAZ27 and FAZ34 caused a reduction in the length of the flagellar pocket.

To look at the flagellar pocket in more detail, the flagellar pocket markers, LmxM.23.0630 and LmxM.06.0030 were used (Sunter et al., 2019). These markers were expressed with a mCherry tag at their C‐terminus in only the parental and null mutants. As expected, LmxM.23.0630 was localised to the bulbous domain, and LmxM.06.0030 was localised to the neck domain in the parental cells (Figure 7c). In both the null mutants, the LmxM.23.0630 signal was still present, yet the signal was now adjacent to the kinetoplast and not distal to it (Figure 7c). However, for LmxM.06.0030, the signal was no longer seen within the flagellar pocket region of both null mutants (Figure 7c), indicating that FAZ27 and FAZ34 loss disrupts the flagellar pocket neck region.

## 3 DISCUSSION

Our screen of *L. mexicana* orthologues of *T. brucei* FAZ proteins identified 27 FAZ proteins in *L. mexicana* with five different FAZ localisation patterns. These distinct localisation classes likely represent the signal from proteins within the different domains of the FAZ—Class 1 consists of proteins likely in the flagellum FAZ domain, Class 2 consists of proteins likely in the cell body FAZ domain running parallel to the flagellum, Class 3 is associated with the flagellar pocket collar region, Class 4 consists of proteins associated with the structures at the flagellum exit point, Class 5 consists of proteins likely in the cell body FAZ domain parallel to the flagellum and flagellar pocket collar region. These localisations are consistent with our initial study of the different FAZ domains in *Leishmania* (Wheeler et al., 2016).

Recently, a study on the Kinesin FAZ7B (not localised in this study) demonstrated another potential localisation, with this protein localising as an asymmetric ring surrounding the majority of the flagellar neck region (Corrales et al., 2021). FAZ3 differs from this, with a much narrower appearance, suggesting that FAZ3 localises to a structure that is distinct from FAZ7B. Despite the complex FAZ structure in *Leishmania*, we have only identified a limited number of localisation patterns. For instance, it appears that only proteins that localise to the linear cell body FAZ domain can also be associated with the horseshoe/ring structure near the collar region. While no proteins localised to both the neck region and the exit point, suggesting these two regions could be separate structures as we hypothesised previously (Wheeler et al., 2016). Nevertheless, it is important to be mindful that the FAZ proteins we identified were limited to proteins conserved in *T. brucei*, and *Leishmania*‐specific proteins that localise to these structures may exist.

The FAZ null mutants had a range of different phenotypes; however, there appeared to be a correlation between the observed phenotypes and their localisation. A similar effect was found in trypanosomes, in which the penetrative power and knockdown phenotype of a FAZ protein often correlated with the proteins' location within the FAZ (Hayes et al., 2014; Lacount et al., 2002; Sunter, Benz, et al., 2015; Sunter & Gull, 2016; Sunter, Varga, et al., 2015; Vaughan et al., 2008; Zhou et al., 2011). The null mutants of FAZ27 and FAZ34, which localised to the flagellum FAZ domain, had cells with shorter cell bodies and flagella, with loose flagella seen in culture. This suggests these FAZ proteins are critical for flagellum attachment and cell morphology. A similar effect was observed for the knockdown of FAZ27 in *T. brucei*; reduction in FAZ and cell body length, changes from trypomastigote to epimastigote morphology and lateral flagellum detachment (An et al., 2020). This shows that proteins within the flagellum and flagellar membrane domains of the FAZ in both species are comparable in terms of hierarchical importance for cell morphology and flagellum attachment.

Proteins that localised to the intracellular FAZ domain had a similar phenotype to those in the flagellum FAZ domain. The deletion of FLABP, which is likely found in the flagellum membrane (Sun et al., 2013) and FAZ5, which is likely located in the cell body membrane (Sunter et al., 2019), resulted in cells with shorter cell bodies and flagella, with loose flagella seen in culture. Deletion of FAZ5 has previously been studied, with the reduction in cell body length and mislocalisation of FLABP::mCherry previously reported; however, loose flagella were not (Sunter et al., 2019). Interestingly, we found that the occurrence of loose flagella and short flagellum cells decreased over time in culture for FAZ27 and FAZ34 null mutants, a similar phenomenon could explain the lack of these phenotypes in the initial FAZ5 null mutant description (Sunter et al., 2019).

Deletion of cell body FAZ domain proteins showed a range of different phenotypes. FAZ2 deletion resulted in cells with a flagellum‐to‐flagellum connection, and we recently showed this was due to the disruption of FAZ, causing an extension of the anterior cell tip that breaks away, forming a membranous structure between the flagella (Halliday et al., 2020). Deletion of CC2D resulted in loose flagella, cells with a short flagellum and a shorter cell body length, in addition to cells with a flagellum‐to‐flagellum connection. This suggests that CC2D has multiple roles, including flagellum attachment and cell tip morphogenesis. Overall, the data from the deletion screen suggests that proteins which localise to the flagellum (FAZ27, FAZ34, FLABP) or are positioned close to the flagellum, such as FAZ5, are required for flagellum attachment, whereas those in the cell body FAZ domain, such as FAZ2 are important for anterior cell tip morphogenesis. Intriguingly, CC2D combines both types of cellular defects, suggesting that this protein is intermediate in its location between FAZ5 and FAZ2.

Our previous work focused on FAZ2 and FAZ5, representing the cell body and intracellular FAZ domains. Here, we investigated in detail the function of FAZ27 and FAZ34, which are present in the flagellum FAZ domain. The loss of either FAZ27 or FAZ34 disrupted the molecular structure of the FAZ, as shown by the change in localisation of FLABP, FAZ5 and FAZ2, with the linear domain of the FAZ within the flagellar pocket neck shorter in these deletion mutants. This shows that proteins present within the flagellum FAZ domain are able to influence the assembly of the cell body and intracellular FAZ domains. Moreover, the flagellum FAZ domain protein FLAM3 was mislocalised in the FAZ27 and FAZ34 null mutants. This shows that recruitment of FLAM3 likely relies on the presence of either FAZ27 or FAZ34, aligning with the data from *T. brucei*, in which FAZ27 and FLAM3 were shown to be interdependent for assembly to the FAZ (An et al., 2020). In addition to defects in the FAZ, we found that there was a reduction in kinetoplast to anterior distance and the mislocalisation of the neck marker in the FAZ27 and FAZ34 null mutants. This further demonstrates the importance of these flagellum FAZ domain proteins for flagellar pocket morphogenesis and organisation. This complements our previous work on the cell body and intracellular FAZ domain proteins, in which deletion of proteins from these domains also disrupted flagellar pocket organisation (Halliday et al., 2020; Sunter et al., 2019), suggesting that loss of proteins in any of the FAZ domains can have a large effect on the pocket itself.

The most striking phenotype that we observed on the deletion of FAZ27 and FAZ34 was the loss of the flagellum, with loose flagella observed not attached to a cell. The reduced length of the FAZ within the flagellar pocket neck likely reduces the attachment area, weakening flagellum attachment; however, the mechanism for the generation of these loose flagella is still unclear. The loose flagella we observed were generally long, suggesting that they are full length or near full length; therefore, if they are formed by a breaking of the flagellum, the break point would be positioned near the proximal end of the flagellum, potentially coincident with the transition zone. An alternative route to generate loose flagella is through the loss of the entire flagellum, including the basal body, from the cell. The presence of flagella with DNA at one end suggest that in some cases the entire flagellum has been uprooted from the cell. It is plausible that both mechanisms contribute to the generation of loose flagella. We found that vortexing resulted in an increase in flagellum loss, suggesting that mechanical stress was a driver of this process. As the loose flagella were generally long this suggests that the longer flagellum may experience more force when it beats than a shorter flagellum. Additionally, longer flagella are older (Wheeler et al., 2011), providing more opportunity for flagellum loss to occur through the accumulation of damage to the FAZ. However, it is possible that short loose flagella were not recovered after centrifugation and so all flagella may be susceptible to detachment. Together, this suggests that an important role for the FAZ is as a force dampener, reducing wave propagation in the flagellum within the flagellar pocket.

Our large‐scale dissection of different components in the different FAZ domains revealed functional groups important for a variety of functions, including FAZ assembly, flagellum attachment and flagellar pocket architecture. This further highlights the importance of the FAZ for the determination of *Leishmania* morphology.

An intriguing phenomenon occurred when we followed the FAZ27 and FAZ34 null mutants in the four‐week period following their initial recovery from transfection. There was an increase in their growth rate, which mirrored a decrease in overall cell body length and the number of short flagellum cells and loose flagella. This suggests that after transfection, the continual growth in culture selects for faster growing variants that have developed compensatory adaptations to support this faster growth rate and reduce the morphology phenotypes. Superficially, the penetrance of the phenotype after deletion of either FAZ27 or FAZ34 was not great; however, the loss of a flagellum and kinetoplast is likely lethal, and this stochastic lethality would provide significant selection pressure for adaptation. This adaptation occurred for two different null mutants with similar changes in phenotype, including a reduction in cell body length and occurring within ~80 cell generations. Such rapid adaptation has been seen in other evolution experiments, including a null mutant analysis in yeast, where the majority of fitness gain occurred within the first 100 generations (Farkas et al., 2022; Helsen et al., 2020; Szamecz et al., 2014). As the loss of FAZ27 and FAZ34 affect the FAZ, it is perhaps unsurprising that they have a similar pattern of changes during this period. However, this suggests there is a common opportunity for a cell to compensate after deleterious perturbation of the FAZ. The likely many close protein–protein interactions involved in FAZ assembly and function suggest that compensatory changes in other interacting and non‐interacting proteins may well be expected in this system. The exact nature of these changes has still to be identified, and whether they result from genomic and/or transcriptomic changes (Targa et al., 2021). Work in yeast showed that there was a preference for changes to genes functionally related to the deleted gene, so there is the potential that these mutants harbour changes in genes important for FAZ assembly and cell morphogenesis (Farkas et al., 2022; Helsen et al., 2020; Szamecz et al., 2014; Venkataram et al., 2016). It is unlikely that such clear changes would be picked up in mutants without complex phenotypes, and cytoskeletal mutants may be particularly amenable to this analysis.

Given the lack of an inducible RNAi system and the priority given to functional analysis through the generation of null mutants in *Leishmania*, we, as a field, need to be more aware of this phenomenon and consider its implications when we interpret the cellular phenotype of null mutants.

## 4 EXPERIMENTAL PROCEDURES

### 4.1 Bioinformatics

TrypTag, the *T. brucei* genome‐wide protein localisation resource, was used to identify proteins with FAZ localisations by interrogating with the search term ‘flagellum attachment zone’; the database was searched during January 2020 (Billington et al., 2023). OrthoMCL embedded in TriTrypDB was used to identify FAZ orthologues in *L. mexicana* (Aslett et al., 2010). The InterPro search tool was used to identify known functional domains (Mitchell et al., 2019).

### 4.2 Generation of protein tagging and deletion constructs

FAZ orthologues in *L. mexicana* were endogenously tagged at either N or C‐terminus. LeishGEdit was used to design primer sequences for use with puro‐mCherry‐puro and blast‐mNeonGreen‐blast pPLOT plasmids for tagging and pT plasmids (pTBlast, pTNeo) for deletion (Beneke et al., 2017). Tagging and deletion amplicons and guide templates were generated as previously described (Beneke et al., 2017). Amplicons were precipitated with sodium acetate‐100% ethanol and washed with 70% ethanol before resuspending the pellet in 20 μL sterile ddH_2_0 prior to transfection.

### 4.3 Generation of FAZ addback plasmids

The open reading frames of LmxM.04.0890 (FAZ27) and LmxM.18.1440 (FAZ34) were amplified by PCR with primers which contained *Xba*l and *Bam*HI restriction sites. The PCR products were digested with *Xba*l and *Bam*HI and ligated into the plasmid pJ1364, which had previously been digested with *Xba*l and *Bam*HI. 10 μg of each plasmid was linearized with *Pac*I, precipitated with sodium acetate‐100% ethanol and washed with 70% ethanol before resuspending with 20 μL sterile ddH_2_0 prior to transfection.

### 4.4 *Leishmania mexicana* cell culture

*Leishmania mexicana* promastigote cells containing were grown in M199 medium with Earle's salts, L‐glutamine, 10% FCS, 40 mM HEPES‐NaOH (pH 7.3), 26 mM NaHCO_3_ and 5 μg mL^−1^ hemin at 28°C. Logarithmic growth was maintained by regular sub‐culturing and cell counts performed with a Beckman Coulter Counter.

The tagging, deletion and add‐back constructs were transfected into 1 × 10^7^ cells resuspended in transfection buffer (200 mM Na_2_HPO_4_, 70 mM NaH_2_PO_4_, 15 mM KCl, 150 mM HEPES (pH 7.3) and 1.5 mM CaCl_2_), using programme X‐001 on an Amaxa Nucleofector IIb. After electroporation, cells were transferred into 10 mL of M199 and incubated at 28°C. After 5–6 h, transfected cells were selected with the appropriate drug (puromycin, 20 μg mL^−1^; blasticidin, 5 μg mL^−1^; G418, 20 μg mL^−1^; phleomycin, 25 μg mL^−1^) and incubated at 28°C for 5–14 days before sub‐culturing of successful transformants. To confirm the loss of the target gene in null mutant cell lines, gDNA was extracted using DNeasy Blood & Tissue kit. Primers were designed to amplify a 400–500 bp region of the target gene.

### 4.5 Light microscopy

Cells between 1 × 10^6^/mL and 1 × 10^7^/mL were either imaged directly from culture or harvested by centrifugation at 1000 × g for 3 min. After supernatant removal, the cells were washed in 1 mL DMEM with Hoechst 33342 (1 μg mL^−1^) and then washed in 1 mL PBS and re‐centrifuged two times before they were resuspended in 20–150 μL PBS, depending on cell number. 2.4 μL was transferred onto a microscope slide and was mounted with a coverslip. The cells were observed on a Zeiss imager Z2 fluorescence microscope with ORCA Flash4 camera and ×63 or ×40 oil immersion objective. mCherry (mCh), GFP and DAPI channels were used to visualise the marker, mNeonGreen (mNG) tagged protein and DNA in kinetoplast/nucleus respectively. The images were captured using Zen Blue software and analysed using Image J.

Cell measurements were made using the line tool in ImageJ (Schneider et al., 2012). The flagellum length was measured from the anterior cell tip to the distal flagellar tip. The cell body length was measured from the posterior cell tip to the anterior cell tip. The anterior end region was measured from the position of the kinetoplast to the anterior cell tip. The mean and standard deviation were calculated for each cell line. Statistical significance length changes were evaluated with one‐way ANOVA, Kruskal–Wallis or Mann–Whitney test.

### 4.6 Flagellum detachment assay

One millilitre of cells in culture medium at a density of 1 × 10^6^/mL–1 × 10^7^/mL was vortexed continuously for 30 s at max speed. Cells were observed, and images were captured on a Zeiss imager Z2 microscope with ORCA Flash4 camera and ×40 oil immersion objective.

## AUTHOR CONTRIBUTIONS

**Jack D. Sunter:** Conceptualization; writing – original draft; formal analysis; supervision; funding acquisition; methodology; project administration. **Clare Halliday:** Investigation; methodology; formal analysis. **Laryssa Vanessa de Liz:** Writing – original draft; formal analysis; visualization. **Sue Vaughan:** Funding acquisition; writing – original draft; supervision; project administration.

## Supporting information


**Table S1.**. FAZ proteins in *Trypanosoma brucei*. Gene IDs and protein information, including predicted molecular weight (MW), TMHMM (transmembrane predictions) and PFAM domains were obtained from TriTrypDB and InterPro (Aslett et al., 2010; Blum et al., 2021). *Leishmania mexicana* orthologues were identified by OrthoMCL in January 2020 (Fischer et al., 2011). †Tb927.9.13880 gene is currently annotated Tb927.9.13860. References for proteins confirmed to be localised in the FAZ of *T. brucei* are shown.
**TABLE S2.** FAZ proteins in *Leishmania mexicana*. Proteins with confirmed localisation in *L. mexicana* FAZ are shown according to their localisation pattern. Orthologues in *Trypanosoma brucei* and their respective localisation in *T. brucei* FAZ are indicated.
**TABLE S3.** Proteins with non‐FAZ localisation. Proteins without localisation in *Leishmania mexicana* FAZ are shown according to their localisation pattern. Orthologues in *Trypanosoma brucei* and their respective localisation in *T. brucei* FAZ are indicated.
**FIGURE S1.** Classification of FAZ proteins in *Trypanosoma brucei* based on their localisation patterns.
**FIGURE S2.** FAZ proteins in *Leishmania mexicana* are classified into five classes based on their localisation patterns.
**FIGURE S3.** *Leishmania* FAZ proteins with complex FAZ localisations.
**FIGURE S4.** Examples of *Leishmania mexicana* orthologs that do not localise to the FAZ.
**FIGURE S5.** Diagnostic PCR confirmed deletion of *Leishmania mexicana* FAZ genes.
**FIGURE S6.** Cell cycle and morphological analysis of FAZ gene deletions in *Leishmania mexicana*.
**FIGURE S7.** FLABP::mCh localisation was not disrupted in specific FAZ null mutants.

## Data Availability

The data that support the findings of this study are available from the corresponding author upon reasonable request.
