# Supplementary material for: Disruption of Leishmania flagellum attachment zone architecture causes flagellum loss
Source: Mol Microbiol. 2023 Nov 27;121(1):53–68. doi: 10.1111/mmi.15199 (PMC10953051; doi:10.1111/mmi.15199)
Supplement: Supplementary file 1 — Table S1.. FAZ proteins in Trypanosoma brucei. Gene IDs and protein information, including predicted molecular weight (MW), TMHMM (transmembrane predictions) and PFAM domains were obtained from TriTrypDB and InterPro (Aslett et al., 2010; Blum et al., 2021). Leishmania mexicana orthologues were identified by OrthoMCL in January 2020 (Fischer et al., 2011). †Tb927.9.13880 gene is currently annotated Tb927.9.13860. References for proteins confirmed to be localised in the FAZ of T. brucei are shown. TABLE S2. FAZ proteins in Leishmania mexicana. Proteins with confirmed localisation in L. mexicana FAZ are shown according to their localisation pattern. Orthologues in Trypanosoma brucei and their respective localisation in T. brucei FAZ are indicated. TABLE S3. Proteins with non‐FAZ localisation. Proteins without localisation in Leishmania mexicana FAZ are shown according to their localisation pattern. Orthologues in Trypanosoma brucei and their respective localisation in T. brucei FAZ are indicated. FIGURE S1. Classification of FAZ proteins in Trypanosoma brucei based on their localisation patterns. FIGURE S2. FAZ proteins in Leishmania mexicana are classified into five classes based on their localisation patterns. FIGURE S3. Leishmania FAZ proteins with complex FAZ localisations. FIGURE S4. Examples of Leishmania mexicana orthologs that do not localise to the FAZ. FIGURE S5. Diagnostic PCR confirmed deletion of Leishmania mexicana FAZ genes. FIGURE S6. Cell cycle and morphological analysis of FAZ gene deletions in Leishmania mexicana. FIGURE S7. FLABP::mCh localisation was not disrupted in specific FAZ null mutants. [file MMI-121-53-s001.pdf]

Disruption of *Leishmania* flagellum attachment zone architecture causes flagellum loss

Supplementary information

Figure S1: Classification of FAZ proteins in *Trypanosoma brucei* based on their localisation patterns.

Figure S2: FAZ proteins in *Leishmania mexicana* are classified into five classes based on their localisation patterns.

Figure S3: *Leishmania* FAZ proteins with complex FAZ localisations.

Figure S4: Examples of *L. mexicana* orthologs that do not localise to the FAZ.

Figure S5: Diagnostic PCR confirmed deletion of *L. mexicana* FAZ genes.

Figure S6: Cell cycle and morphological analysis of FAZ gene deletions in *L. mexicana*.

Figure S7: FLABP::mCh localisation was not disrupted in specific FAZ null mutants.

Table S1: FAZ proteins in *Trypanosoma brucei*.

Table S2: FAZ proteins in *Leishmania mexicana*.

Table S3: Proteins with non-FAZ localisation.

Figure S1

Type of FAZ localisation	No. of genes in *T. brucei*	Example image
**Full length**	**23**	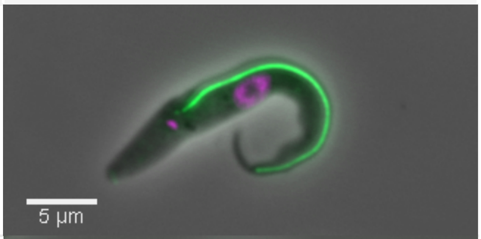
**Full length-distal enriched**	**18**	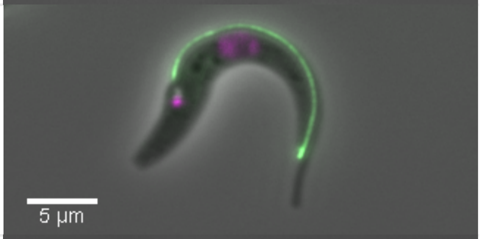
**Full length-proximal enriched**	**3**	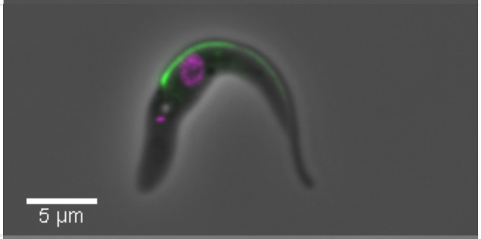
**Distal only**	**17**	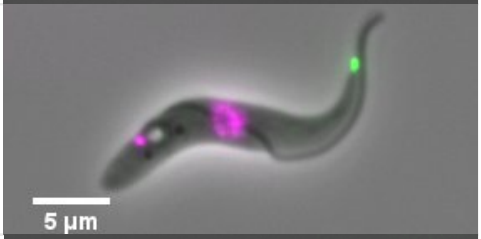
**Proximal only**	**3**	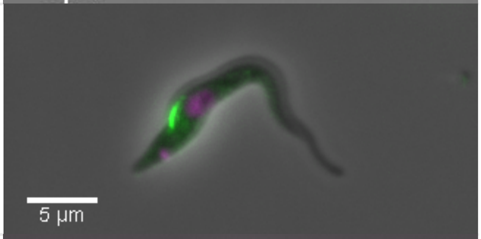
**FAZ-ER**	**9**	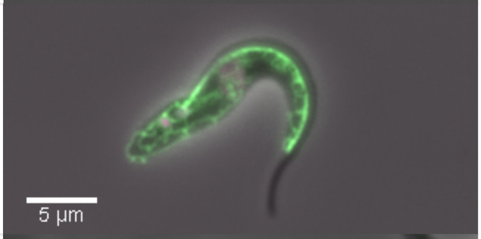
**Complex**	**23**	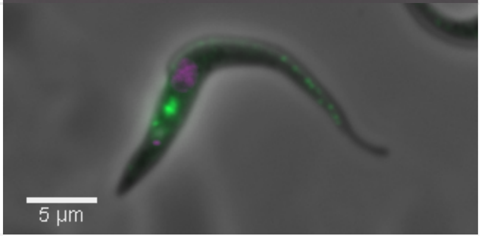

Figure S1: Classification of FAZ proteins in *Trypanosoma brucei* based on their localisation patterns. All proteins were tagged with mNeonGreen as part of the TrypTag project and assigned to categories based on their localisation patterns. Categories: Full length (example: FAZ8, Tb927.4.2060), Full length-distal enriched (example FAZ2, Tb927.1.4310), Full length-proximal enriched (example: FAZ19, Tb927.3.3300), Distal only (FAZ21, Tb927.7.5240), Proximal only (hypothetical protein, Tb927.9.8240), FAZ-ER (reticulon domain protein, Tb927.6.3840), Complex (Autophagy-related protein 27, Tb927.6.3940 with endocytic/FAZ signal). Example images are kindly provided by TrypTag.

Figure S2

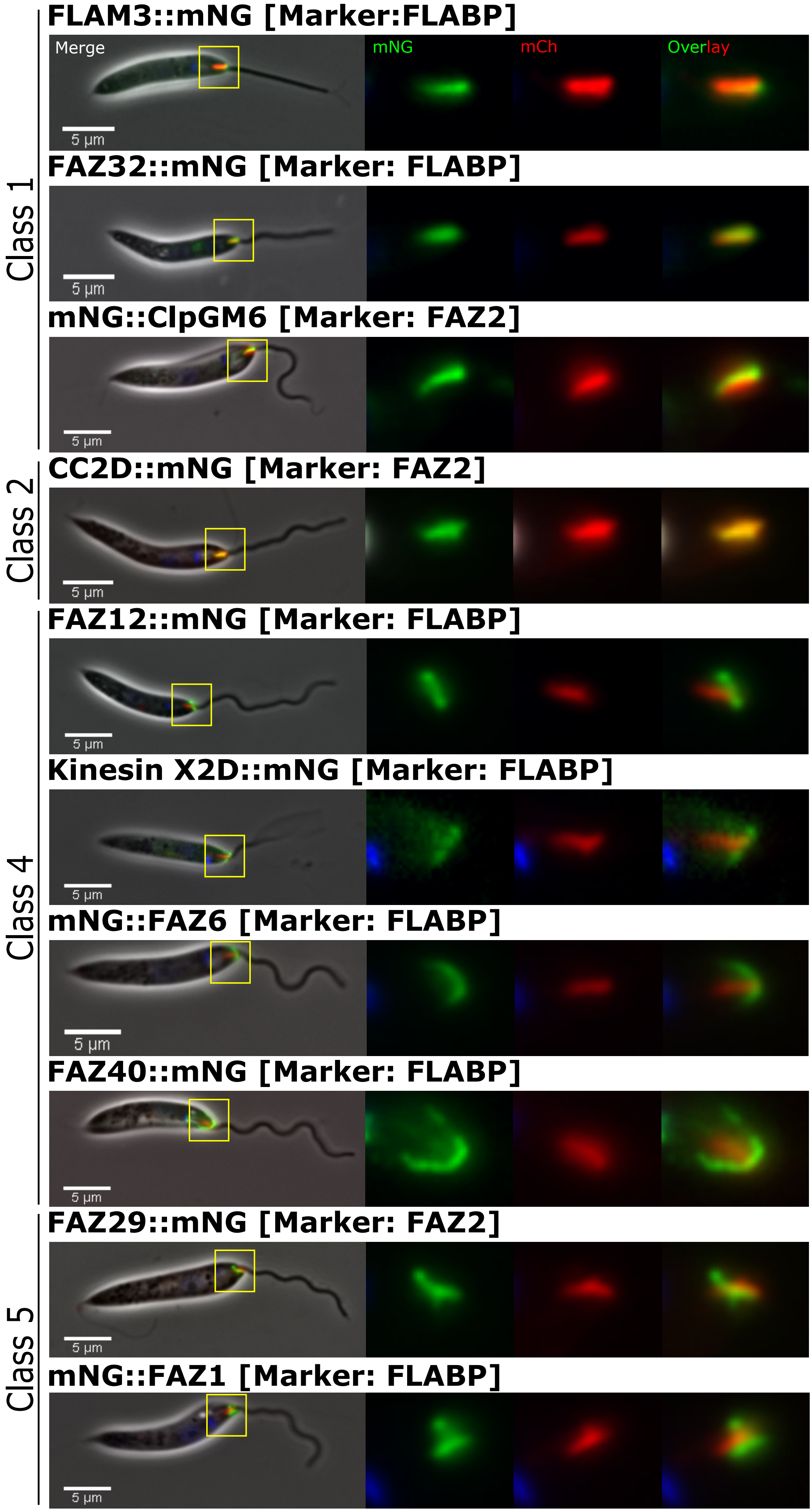

Figure S2: FAZ proteins in *Leishmania mexicana* are classified into five classes based on their localisation patterns. Class 1 - FLAM3 and FAZ32 co-localised with FLABP, while ClpGM6 localised adjacent to FAZ2. Class 2 - CC2D co-localised with FAZ2.

Figure S2

Class 4 - FAZ12, Kinesin X2D, FAZ6 and FAZ40 were localised at the flagellum exit point. Class 5 - FAZ29 and FAZ1 localised to the FAZ within the cell body and the collar region. From left, an overlay of the phase contrast (grey), mNeonGreen (mNG) tagged protein (green), mCherry (mCh) tagged marker (red) and Hoechst DNA (blue) then mNG only, and mCh only, the far right is an overlay of mNG and mCh. Scale 5 µm. Other FAZ proteins are shown in Figure 2.

Figure S3

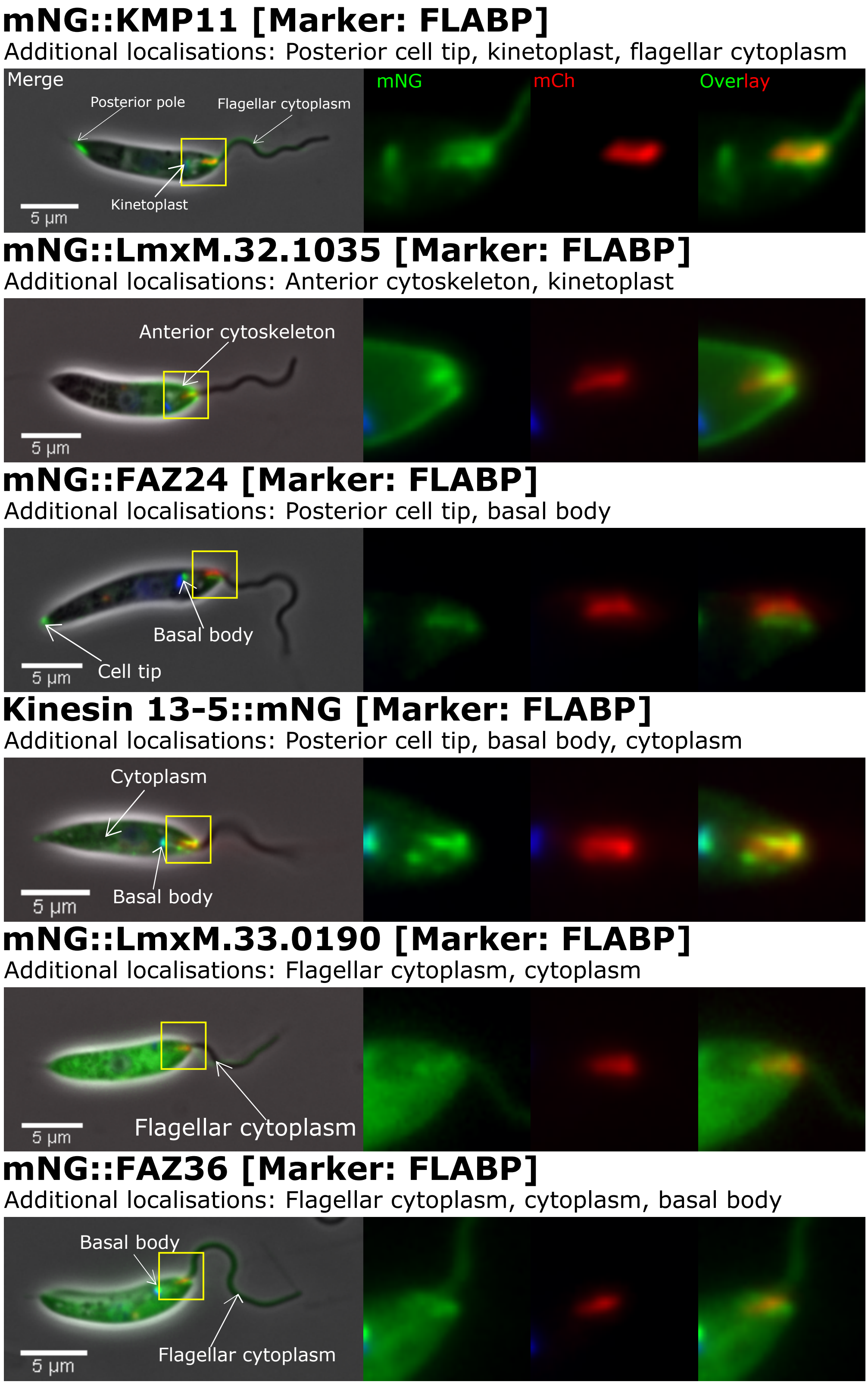

Figure S3: *Leishmania* FAZ proteins with complex FAZ localisations. Widefield fluorescence images for each FAZ protein in *L. mexicana*, with a complex localisation pattern. From left, an overlay of the phase contrast (grey), mNeonGreen (mNG) tagged protein (green), mCherry (mCh) tagged marker (red) and Hoechst DNA (blue), scale 5 µm. Then enlarged images of the FAZ region with mNG only, mCh only, and an overlay of mNG, mCh, and DNA in the far right. Additional localisations are indicated beneath protein names, with arrows indicating specific features.

Figure S4

mNG::LmxM.31.2610 [Marker: FLABP]
Posterior cell tip, nucleus

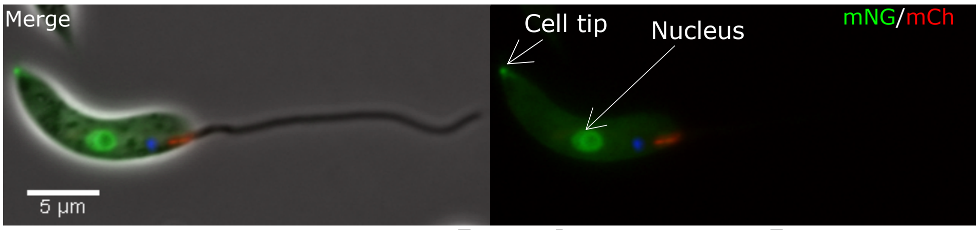

LmxM.34.3720::mNG [Marker FLABP]
Posterior cytoskeleton

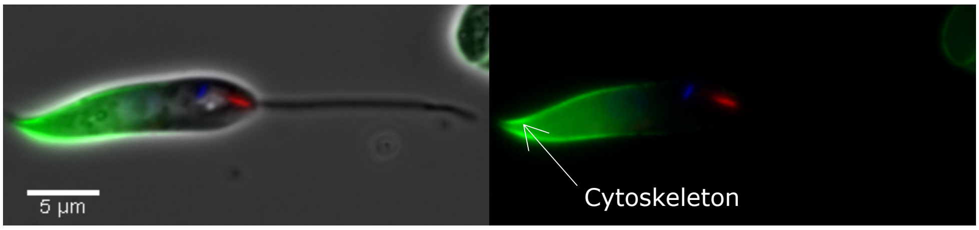

mNG::LmxM.11.1320 [Marker FLABP]
Endoplasmic reticulum

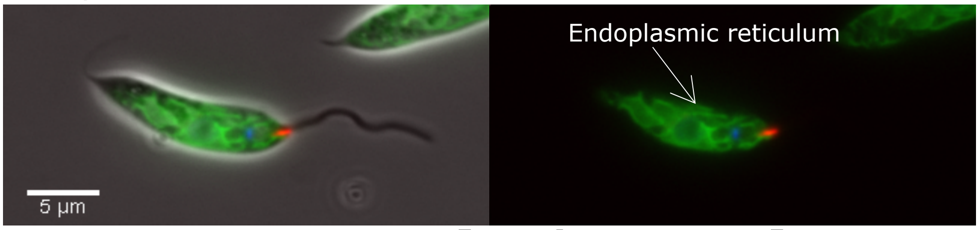

mNG::LmxM.19.0140 [Marker FLABP]
Lysosome, kinetoplast

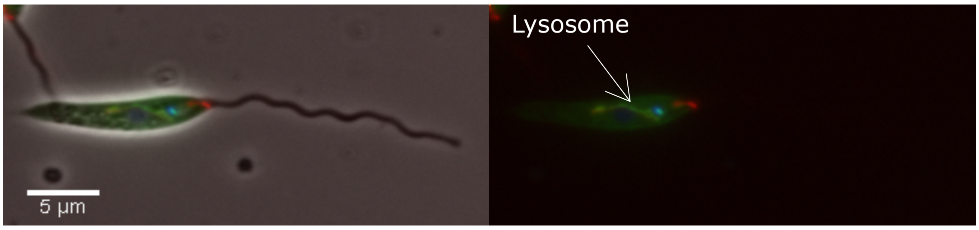

Figure S4: Examples of *L. mexicana* orthologs that do not localise to the FAZ. Widefield fluorescence images of example proteins that do not localise to the FAZ in *L. mexicana*. From left, an overlay of the phase contrast (grey), mNeonGreen (mNG) tagged protein (green), mCherry (mCh) tagged marker (red) and Hoechst DNA (blue), scale 5 µm. Then enlarged images of the FAZ region with mNG only, mCh only, and an overlay of mNG, mCh, and DNA in the far right. Annotations are displayed with arrows indicating specific features.

Figure S5

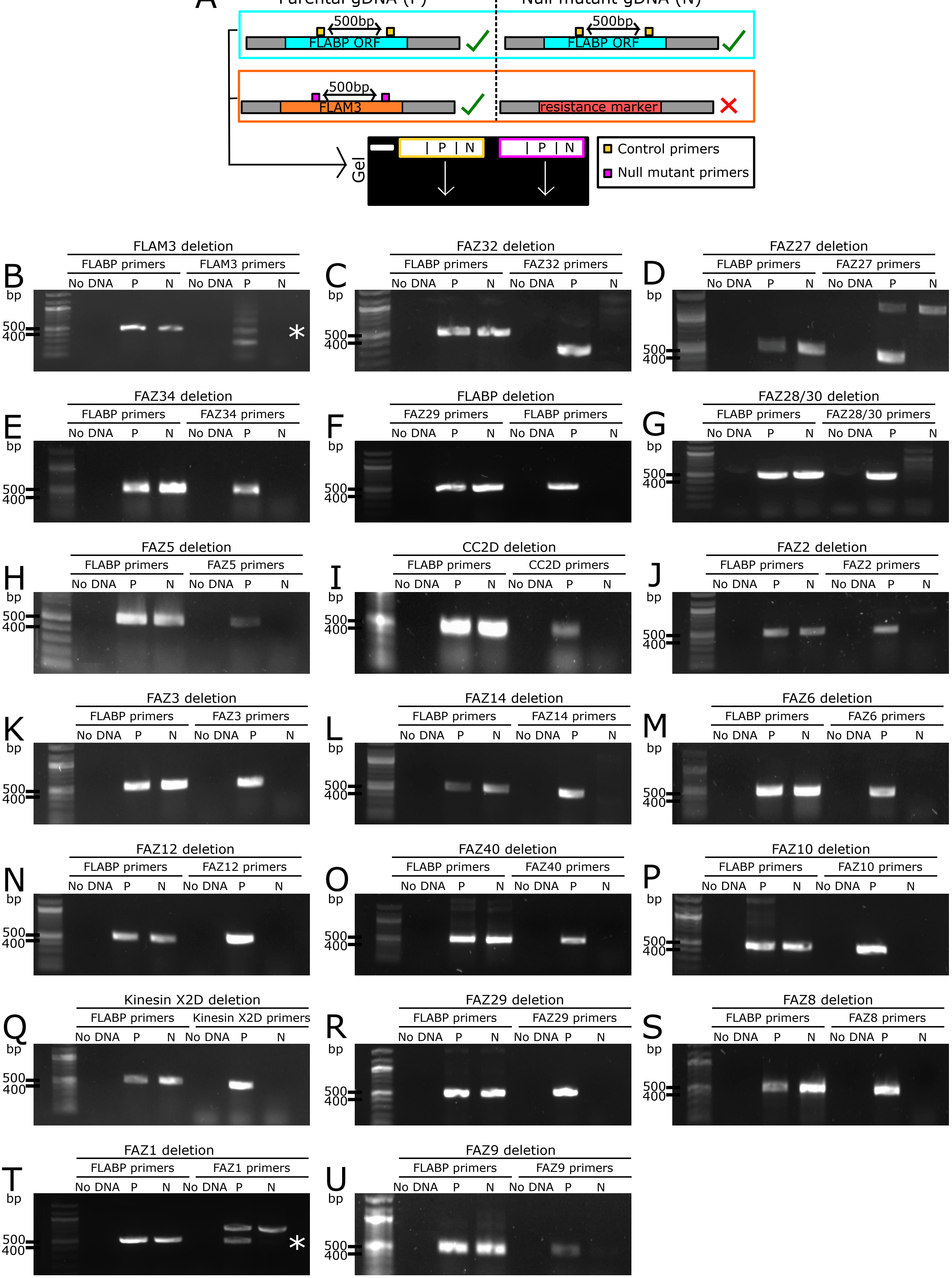
A
Parental gDNA (P)
Null mutant gDNA (N)
500bp
FLABP ORF
FLAM3
resistance marker
Gel
P | N
Control primers
Null mutant primers
B FLAM3 deletion
FLABP primers
FLAM3 primers
C FAZ32 deletion
FAZ32 primers
D FAZ27 deletion
FAZ27 primers
E FAZ34 deletion
FAZ34 primers
F FLABP deletion
FAZ29 primers
G FAZ28/30 deletion
FAZ28/30 primers
H FAZ5 deletion
FAZ5 primers
I CC2D deletion
CC2D primers
J FAZ2 deletion
FAZ2 primers
K FAZ3 deletion
FAZ3 primers
L FAZ14 deletion
FAZ14 primers
M FAZ6 deletion
FAZ6 primers
N FAZ12 deletion
FAZ12 primers
O FAZ40 deletion
FAZ40 primers
P FAZ10 deletion
FAZ10 primers
Q Kinesin X2D deletion
Kinesin X2D primers
R FAZ29 deletion
S FAZ8 deletion
FAZ8 primers
T FAZ1 deletion
FAZ1 primers
U FAZ9 deletion
FAZ9 primers
No DNA P N
bp
500
400
*

Figure S5

Figure S5: Diagnostic PCR confirmed deletion of *L. mexicana* FAZ genes. A) Representative schematic for the confirmation of *FLAM3* deletion; B) *FLAM3*, C) *FAZ32*, D) *FAZ27*, E) *FAZ34*, F) *FLABP*, G) *FAZ28/30*, H) *FAZ5*, I) *CC2D*, J) *FAZ2*, K) *FAZ3*, L) *FAZ14*, M) *FAZ6*, N) *FAZ12*, O) *FAZ40*, P) *FAZ10*, Q) *Kinesin X2D*, R) *FAZ29*, S) *FAZ8*, T) *FAZ1*, and U) *FAZ9*. gDNA was extracted from null mutants and tested alongside the parental gDNA for confirmation of gene deletion. LmxM.10.0620 (FLABP) ORF was used as a positive control for amplification except for the FLABP null mutant, for which FAZ29 ORF was amplified. The primers were designed to amplify a 500 bp region from the ORF except for FAZ32, in which a 400 bp region was amplified. P: Parental cell line. N: Null mutant cell line.

Figure S6

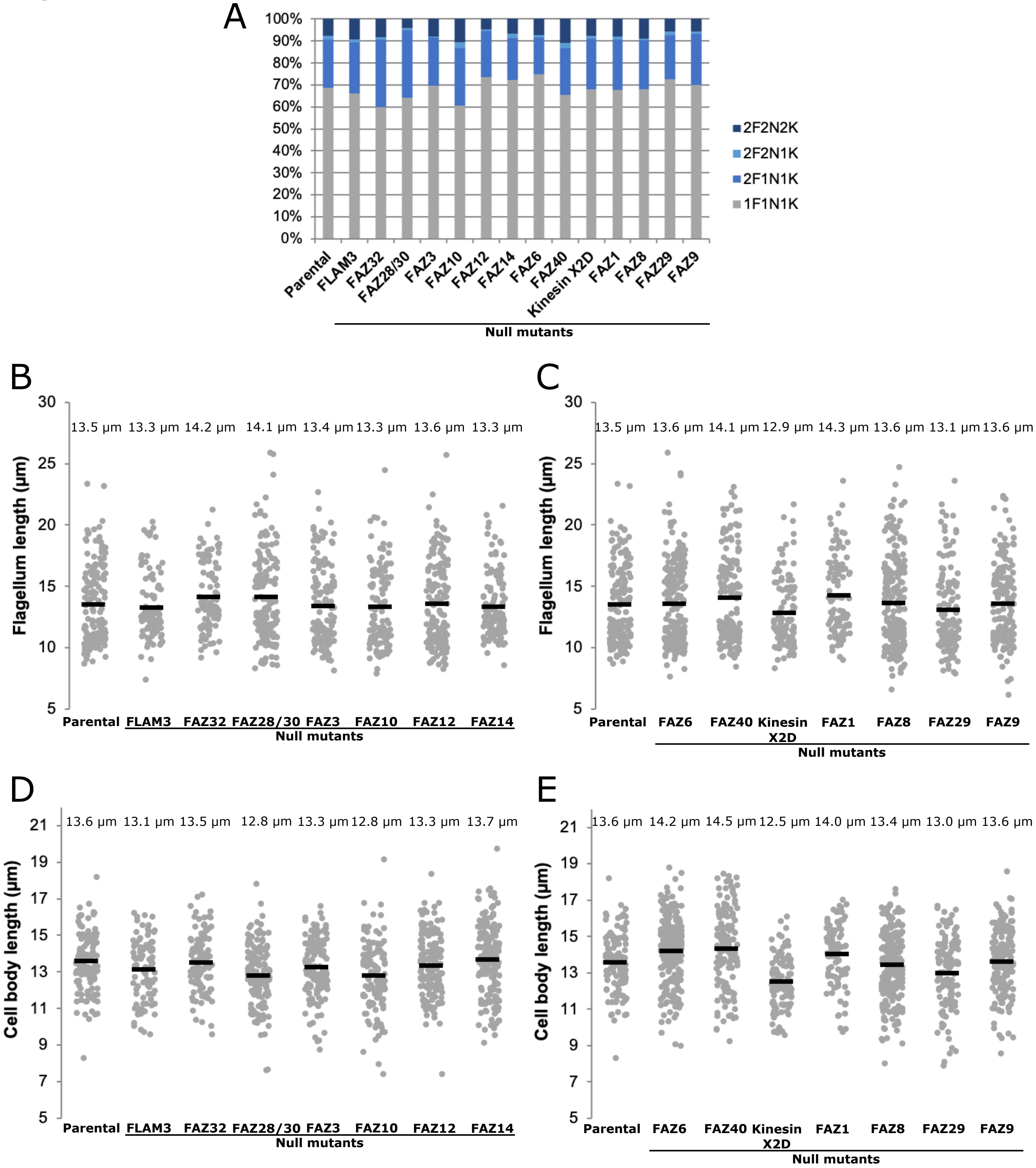

Figure S6: Cell cycle and morphological analysis of FAZ gene deletions in *L. mexicana*. A) Cell cycle quantitation in parental and the null mutants of FLAM3, FAZ32, FAZ28/30, FAZ3, FAZ10, FAZ12, FAZ14, FAZ6, FAZ40, Kinesin X2D, FAZ1, FAZ8, FAZ29, and FAZ9. Cell cycle stages include 1F1N1K, 2F1N1K, 2F2N1K, and 2F2N2K. Percentages were calculated from ≥100 cells. B-E) Length of flagellum (B, C) and cell body (D, E) of parental and the null mutants of FLAM3, FAZ32, FAZ28/30, FAZ3, FAZ10, FAZ12, FAZ14, FAZ6, FAZ40, Kinesin X2D, FAZ1, FAZ8, FAZ29, and FAZ9. Each dot represents the length measurement of an individual cell, and the mean (black bar) was calculated from these length measurements (≥86 cells).

Figure S7

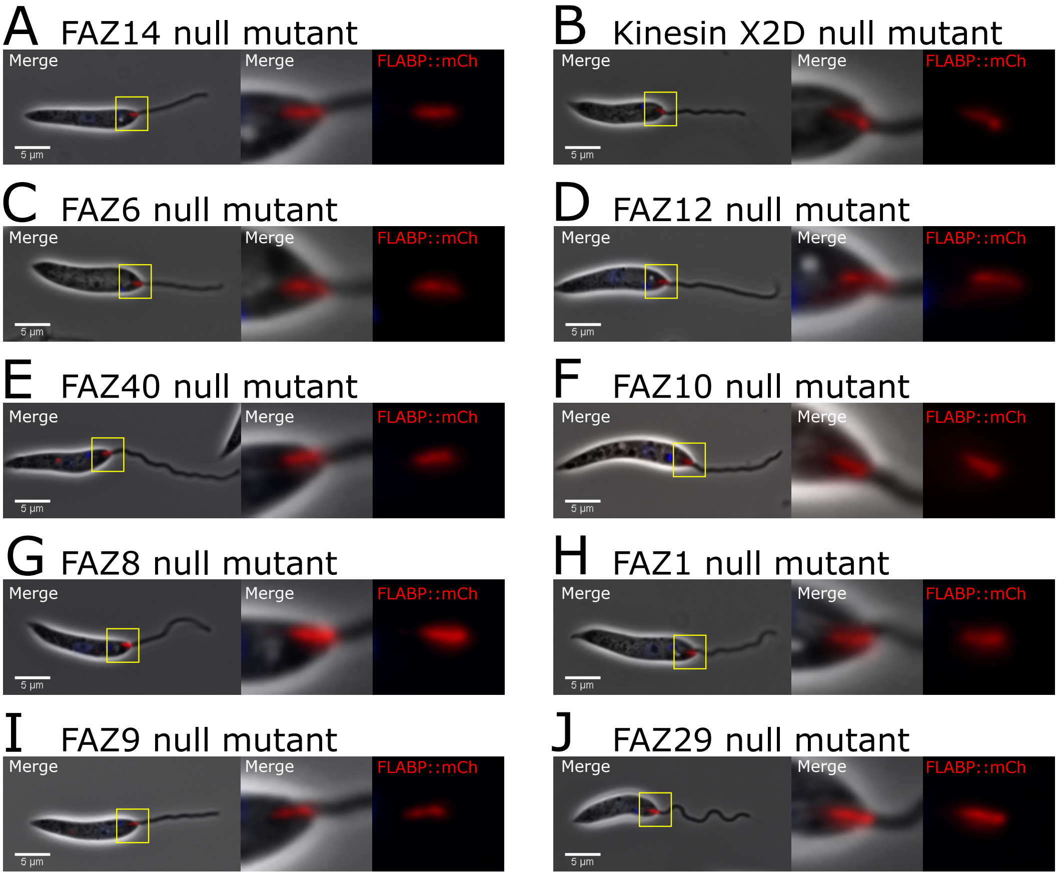

Figure S7: FLABP::mCh localisation was not disrupted in specific FAZ null mutants. FAZ14, Kinesin X2D, FAZ6, FAZ12, FAZ40, FAZ10, FAZ8, FAZ1, FAZ9, and FAZ29 null mutants. All of the cell lines expressed the FLABP::mCh marker. Images of cells (merge) contain phase (grey), mCh (red) and Hoechst 33342 (blue), scale 5 µm. The enlarged FAZ region on the right shows merge or mCh (red) and DNA only.

Figure S8

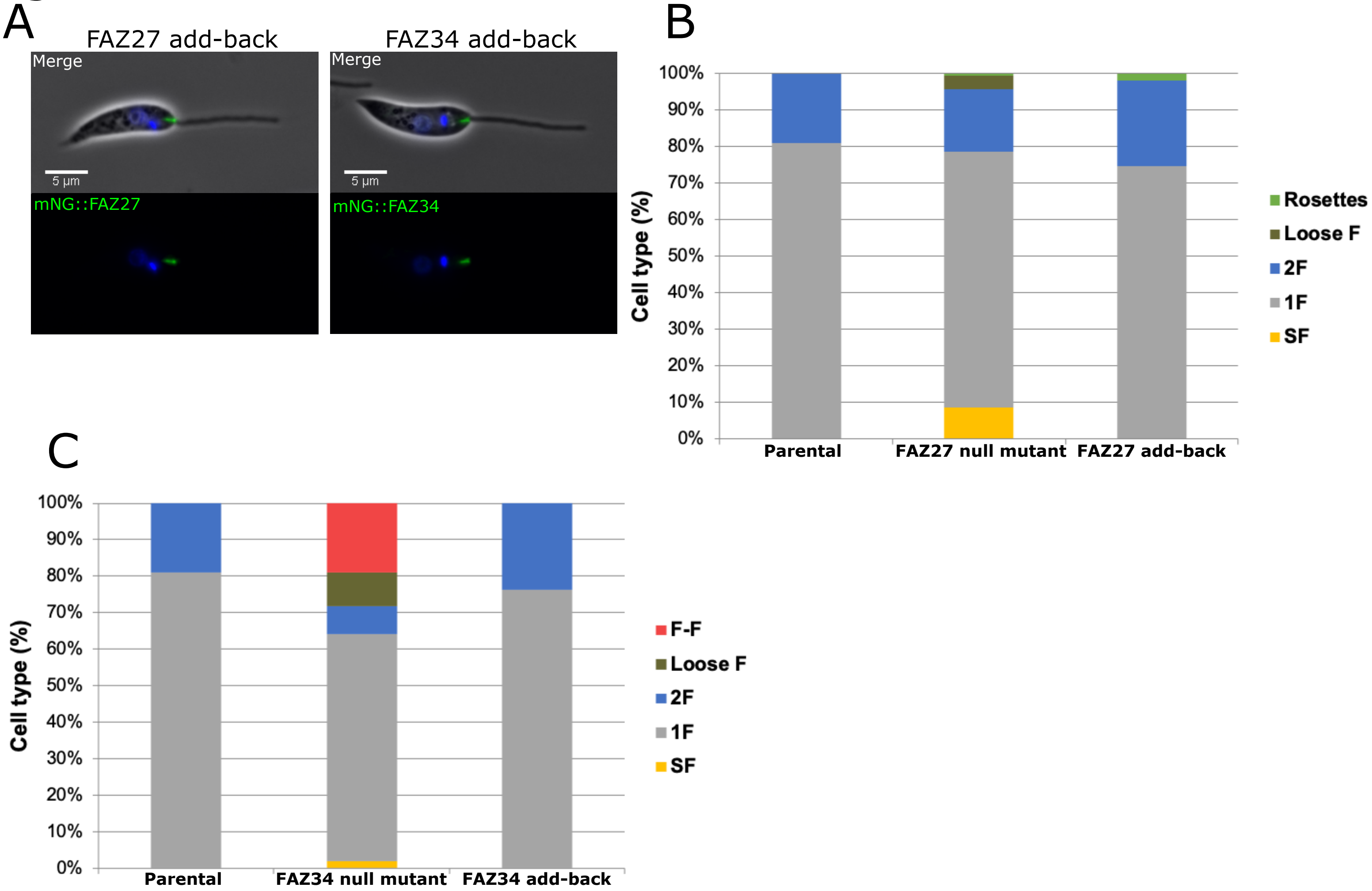

Figure S8: Add back of FAZ27 and FAZ34 protein restored parental phenotype. A) FAZ27 and FAZ34 add-back cells expressing FAZ protein tagged with mNG. FAZ27 and FAZ34 localised to a short line in the FAZ region. B) Quantitation of cell types observed in parental, FAZ27 null mutant and FAZ27 add-back in week 4. C) Quantitation of cell types observed in parental, FAZ34 null mutant and FAZ34 addback in week 4. Each experiment (cell n>90) was performed in duplicate, and the representative data from one experiment is shown. Loose F: loose flagellum. SF: short flagellum. 2F: two flagella. 1F: one flagellum.

Table S1: FAZ proteins in *Trypanosoma brucei* . Gene IDs and protein information, including predicted molecular weight (MW), TMHMM (transmembrane predictions) and PFAM domains were obtained from TriTrypDB and InterPro (Aslett et al., 2009; Blum et al., 2021). *L. mexicana* orthologs were identified by OrthoMCL in January 2020 (Fischer et al., 2011). †Tb927.9.13880 gene is currently annotated Tb927.9.13860. References for proteins confirmed to be localised in the FAZ of *T. brucei* are shown.

Gene ID	Protein name	MW	TMHMM	PFAM domains	*L. mexicana* ortholog	Reference
**Full Length**						
Tb927.9.8650	Flagellum attachment zone protein	12,29	no		LmxM.04.1100	this work
Tb927.8.4780	Flagellar Member 3 (FLAM3)	468,13	no	Clustered mitochondria; Translation initiation factor eIF3	LmxM.16.1660	(Rotureau et al., 2014)
Tb927.11.1090	Calpain-like protein, putative (ClpGM6)	662,98	no	Peptidase C2, calpain, catalytic domain x2	LmxM.27.0490	(Hayes et al., 2014)
Tb927.11.1110	Calpain, putative	171,46	no	Peptidase C2, calpain, catalytic domain; Calpain large subunit, domain III superfamily	LmxM.27.0490	
Tb927.10.8830	Flagellum attachment zone protein 5	66,66	7	Chaperone J-domain Chaperone J-domain	LmxM.36.5970	(Sunter et al., 2015)
Tb927.4.2080	C2 domain containing protein	104,88	no	C2 domain	LmxM.33.2540	(Zhou et al., 2011)
Tb927.8.7420	Flagellum attachment zone protein 30	96,39	no	Camp-Binding Protein 1-related; Sav2460 like domain	LmxM.30.2590	this work
Tb927.11.12530	Flagellum attachment zone protein	90,65	no		LmxM.09.0520	(Sunter et al., 2015)
Tb927.6.840	Flagellum attachment zone protein 29	38,16	no	Glutathione S-transferase N-terminal domain	LmxM.12.0360	this work
Tb927.4.3740	Flagellar attachment zone protein 1	192,57	no		LmxM.33.0690	(Vaughan et al., 2008)
Tb927.10.14320	Flagellum attachment zone protein	121,80	no	Armadillo-type fold	LmxM.31.0140	(Sunter et al., 2015)
Tb927.4.2060	Flagellum attachment zone protein 8	66,84	no		LmxM.33.2570	(Zhou et al., 2015; Sunter et al., 2015)
Tb927.9.9320	MAPK/MAK/MRK overlapping kinase, putative	41,76	no	Protein kinase domain	LmxM.34.5010	
Tb927.10.9700	Flagellum attachment zone protein	132,58	no	C2 domain x3	LmxM.36.4330	(Zhou et al., 2018a)
Tb927.4.5340	Flagellum attachment zone protein	94,70	no		LmxM.30.3110	(Morriswood et al., 2013)

Tb927.3.4710	Flagellum attachment zone protein	59,75	no			(Zhou et al., 2018a)
Tb927.9.2075	Flagellum attachment zone protein	302,50	no			this work
Tb927.10.2880	Flagellum attachment zone protein	303,77	22	Ion transport protein x4		(Oberholzer et al., 2011)
Tb927.10.5870	Flagellum attachment zone protein	49,47	no			(Zhou et al., 2018a)
Tb927.10.7210	Flagellum attachment zone protein	26,26	no			(McAllaster et al., 2015)
Tb927.10.11360	Flagellum attachment zone protein 33	73,25	no	WD40/YVTN repat-like containing domain superfamily, WD40-repeat-containing domain superfamily, protein phosphatase 2A regulatory subunit PR55		this work
Tb927.11.12870	Hypothetical protein, conserved	78,40	11			
**Full length - Distal enriched**						
Tb927.8.4050	FLA1-binding protein	83,06	2	YWTD domain	LmxM.10.0620	(Sun et al., 2013)
Tb927.10.12630	Flagellum attachment zone protein 34	25,14	no	EF-hand domain pair & EF-hand domain	LmxM.18.1440	this work
Tb927.1.4310	Flagellum attachment zone protein 2	183,76	no		LmxM.12.1120	(Zhou et al., 2015; Sunter et al., 2015)
Tb927.9.8350	Flagellum attachment zone protein	66,85	no		LmxM.04.0890	(An et al., 2020)
Tb927.4.5000	Flagellum attachment zone protein	96,39	no	TerD domain x2	LmxM.30.2590	this work
Tb927.11.2590	Flagellum attachment zone protein	121,14	no		LmxM.32.2460	(Hu, Zhou and Li, 2015b)
Tb927.7.3330	Flagellar attachment zone protein	502,65	no		LmxM.22.1320	(Morriswood et al., 2013)
Tb927.8.6980	Flagellum attachment zone protein	95,06	no		LmxM.30.3110	(Hu, Zhou and Li, 2015b)
Tb927.10.840	Flagellum attachment zone protein	195,48	no	WD domain, G-beta repeat x2	LmxM.21.1240	(Sunter et al., 2015)
Tb927.9.10530	Flagellum attachment zone protein	118,72	no			(Sunter et al., 2015)
Tb927.3.1020	Flagellum attachment zone protein	54,34	no			(Hu, Zhou and Li, 2015b)
Tb927.8.7070	Flagellum attachment zone protein	35,43	no			(McAllaster et al., 2015)
Tb927.5.3460	Flagellum attachment zone protein	57,44	no	LysM domain		(McAllaster et al., 2015)
Tb927.9.8180	Flagellum attachment zone protein	70,84	no			this work
Tb927.8.5350	Flagellum attachment zone protein	18,94	no			this work
Tb927.9.8330	Flagellum attachment zone protein	52,61	no			this work
Tb927.11.3400	Flagellum attachment zone protein	34,88	no			this work

Tb927.4.1960	Flagellum attachment zone protein	37,39	no			this work
**Full length - Proximal enriched**						
Tb927.11.2070	Flagellum attachment zone protein	15,31	no		LmxM.27.1400	this work
Tb927.3.3300	Flagellum attachment zone protein	89,20	no			(Zhou, Hu and Li, 2016)
Tb927.1.5030	Flagellum attachment zone protein	80,77	no	Leucine Rich Repeat_1 & 6		this work
**Distal only**						
Tb927.8.6830	Kinesin, putative	198,66	no	Kinesin motor domain	LmxM.24.1430	(Zhou et al., 2018b)
Tb927.10.12470	Flagellum attachment zone protein	58,39	no		LmxM.18.1560	this work
Tb927.2.2360	Ankyrin repeats (3 copies)/Zinc	74,57	no	Ankyrin repeats (3 copies), Zinc	LmxM.02.0140	
Tb927.10.5240	cAMP binding protein, putative	188,21	no	Cyclic nucleotide-binding domain & C2 domain x3	LmxM.36.0830	
Tb927.10.6360	FAZ-tip-localizing protein required for cytokinesis	55,52	no		LmxM.36.1920	(Zhou et al., 2018a)
Tb927.10.8240	Cytokinesis initiation factor 4	42,85	no		LmxM.36.6960	(Hilton et al., 2018)
Tb927.10.15390	Flagellum attachment zone protein	123,56	no	Kinesin motor domain	LmxM.19.0680	(Sunter et al., 2015)
Tb927.11.9290	Flagellum attachment zone protein	85,45	no	Protein kinase domain	LmxM.28.1650	(Zhou, Hu and Li, 2016)
Tb927.11.15800	Tip Of Extending FAZ protein 1	89,59	no		LmxM.31.2610	(Zhou et al., 2016; McAllaster et al., 2015)
Tb927.10.12920	Flagellum attachment zone protein	112,87	no			(Zhou, Hu and Li, 2016)
Tb927.7.5240	Flagellum attachment zone protein	53,49	no			(Zhou et al., 2018a)
Tb927.11.11240	Flagellum attachment zone protein	23,00	no			this work
Tb927.10.13100	Cytokinesis initiation factor 3	50,92	no			(Kurasawa et al., 2018; Zhou et al., 2018a)
Tb927.10.9720	RNA-editing-associated protein 1	64,38	no			
Tb927.11.5410	SUMO-interacting motif-containing protein	70,77	no			
Tb927.6.3710	Hypothetical protein, conserved	78,29	no			
Tb927.10.12670	Hypothetical protein, conserved	58,47	no			
**Proximal only**						
Tb927.4.5330	Flagellum attachment zone protein	97,24	no			this work
Tb927.3.5220	Hypothetical protein, conserved	65,03	no			
Tb927.9.8240	Hypothetical protein, conserved	55,58	no			
**FAZ-ER**						

Tb927.1.4420	ABC transporter, putative	104,68	no	ABC transporter	LmxM.12.1190	
Tb927.6.3840	Reticulon domain protein	21,07	3	Reticulon	LmxM.29.2580	
Tb927.7.3070	UAA transporter family, putative	54,85	8	UAA transporter family	LmxM.22.1010	
Tb927.7.3760	Phosphatidylserine synthase,	57,60	8	Phosphatidyl serine synthase	LmxM.14.1200	
Tb927.11.5370	Hypothetical protein, conserved	76,64	7		LmxM.24.0700	
Tb927.11.6060	Major facilitator superfamily,	51,32	11	Major facilitator superfamily	LmxM.11.1320	
Tb927.11.13230	VAMP-associated protein, putative	23,91	1	MSP (Major sperm protein) domain	LmxM.09.1050	(Lacomble et al., 2012)
Tb927.11.15870	Hypothetical protein, conserved	42,43	2	VASt domain	LmxM.31.2680	
Tb927.10.13740	Flagellum attachment zone protein	67,31	3	SMP domain, C2 domain x2		this work
**Complex**						
Tb927.4.3520	Amastin surface glycoprotein,	19,46	4	Amastin surface glycoprotein	LmxM.16.0490	(Hilton et al., 2018)
Tb927.6.3940	Autophagy-related protein 27,	39,21	3	Autophagy-related protein	LmxM.29.2670	
Tb927.8.2030	Posterior and Ventral Edge protein 1	48,24	no		LmxM.23.0080	
Tb927.9.11540	Hypothetical protein, conserved	50,79	no		LmxM.34.3720	
†Tb927.9.13880	kinetoplastid membrane protein 11-	11,08	no	Kinetoplastid membrane protein	LmxM.34.2221	(Li and Wang, 2008; Li et al.,
Tb927.9.13820	Kinetoplastid membrane protein 11-	11,08	no	Kinetoplastid membrane protein	LmxM.34.2221	(Li and Wang, 2008; Li et al.,
Tb927.10.1230	Enriched in surface-labeled	37,68	5		LmxM.21.0940	
Tb927.10.1620	Phosphoserine/threonine/tyrosine-binding protein, putative	59,94	no	Dual specificity protein phosphatase domain	LmxM.21.0700	
Tb927.10.2480	Hypothetical protein, conserved	105,08	3		LmxM.33.0060	
Tb927.10.2610	Domain of unknown function (DUF1935), putative	54,36	no	Domain of unknown function (DUF1935) x4	LmxM.33.0190	
Tb927.10.720	Flagellum attachment zone protein	118,09	no	PB1 domain	LmxM.21.1350	(Zhou et al., 2018a)
Tb927.10.13010	cAMP-dependent protein kinase catalytic subunit 3 (PKAC3)	39,18	no	Protein kinase domain	LmxM.18.1080	
Tb927.10.14770	Associated kinase of Tb14-3-3	70,53	no	Protein kinase domain, CAP Gly-rich domain	LmxM.19.0140	
Tb927.11.3280	Kinesin-13 5, putative	80,07	no	SAM domain (Sterile alpha motif) & Kinesin motor domain	LmxM.13.1610	
Tb927.10.11650	Hypothetical protein, conserved	17,39	no		LmxM.32.1035	
Tb927.10.870	Furrow 1 protein	151,85	no		LmxM.21.1220	(Zhou et al., 2018a)

Tb927.10.14400	Hypothetical protein, conserved	125,26	1		LmxM.31.0220	
Tb927.11.1640	Stumpy formation signalling pathway protein, putative	42,25	2		LmxM.27.1040	
Tb927.11.3300	Spindle assembly abnormal 4	107,86	no		LmxM.13.1590	(Hu, Zhou and Li, 2015b)
Tb927.11.11480	Trichohyalin, putative	78,97	no			(Zhou et al., 2018a)
Tb927.1.4280	Hypothetical protein, conserved	75,28	no			
Tb927.7.5190	Hypothetical protein, conserved	126,87	no			
Tb927.9.2760	EB1-like C-terminal motif containing protein, putative	57,00	no	EB1 C terminal domain, microtubule associated protein RP/EB, Calponin homology domain		
Tb927.9.14290	Cytokinesis initiation factor 2	49,83	no			(Zhou, Hu and Li, 2016)

Table S2: FAZ proteins in *Leishmania mexicana* . Proteins with confirmed localisation in *L. mexicana* FAZ are shown according to their localisation pattern. Orthologs in *T. brucei* and their respective localisation in *T. brucei* FAZ are indicated.

L. mexicana		***T. brucei***	
**Gene ID**	**Protein name**	**Gene ID**	**Localisation**
**Linnear on flagellum side**			
LmxM.10.0620	FLABP	Tb927.8.4050	Full-distal
LmxM.16.1660	FLAM3	Tb927.8.4780	Full length
LmxM.04.0890	FAZ27	Tb927.9.8350	Full-distal
LmxM.04.1100	FAZ32	Tb927.9.8650	Full length
LmxM.18.1440	FAZ234	Tb927.10.12630	Full-distal
LmxM.27.0490	ClpGM6	Tb927.11.1090 and Tb927.11.1110	Full length
**Linnear on cell body side**			
LmxM.12.1120	FAZ2	Tb927.1.4310	Full-distal
LmxM.33.2540	CC2D	Tb927.4.2080	Full length
LmxM.36.5970	FAZ5	Tb927.10.8830	Full length
LmxM.30.2590	FAZ28/30	Tb927.8.7420 and Tb927.4.5000	Full length
**Ring/horseshoe at collar region**			
LmxM.09.0520	FAZ3	Tb927.11.12530	Full length
**Ring at exit point**			
LmxM.30.3110	FAZ14	Tb927.8.6980 and Tb927.4.5340	Full-distal
LmxM.21.1240	FAZ6	Tb927.10.840	Full-distal
LmxM.32.2460	FAZ12	Tb927.11.2590	Full-distal
LmxM.22.1320	FAZ10	Tb927.7.3330	Full-distal
LmxM.18.1560	FAZ40	Tb927.10.12470	Distal only
LmxM.24.1430	Kinesin X2D	Tb927.8.6830	Distal only
**Linear on cell body side and at collar region**			
LmxM.12.0360	FAZ29	Tb927.6.840	Full length
LmxM.33.2570	FAZ8	Tb927.4.2060	Full length
LmxM.33.0690	FAZ1	Tb927.4.3740	Full length
LmxM.31.0140	FAZ9	Tb927.10.14320	Full length

Complex			
LmxM.34.2221	KMP11	*Tb927.9.13880 and Tb927.9.13820	Complex
LmxM.32.1035	Hypothetical protein	Tb927.10.11650	Complex
LmxM.21.1350	FAZ24	Tb927.10.720	Complex
LmxM.13.1610	Kinesin 13-5	Tb927.11.3280	Complex
LmxM.33.0190	Domain of unknown function (DUF1935), putative	Tb927.10.2610	Complex
LmxM.27.1400	FAZ36	Tb927.11.2070	Full length- Proximal enriched

Table S3: Proteins with non-FAZ localisation. Proteins without localisation in *L. mexicana* FAZ are shown according to their localisation pattern. Orthologs in *T. brucei* and their respective localisation in *T. brucei* FAZ are indicated.

L. mexicana			*T. brucei*	
**Gene ID**	**Protein name**	**Localisations**	**Gene ID**	**Localisation**
	**Cytoplasm**			
LmxM.12.1190	ABC transporter	Cytoplasm (weak)	Tb927.1.4420	FAZ-ER
LmxM.34.5010	MPK13	Cytoplasm reticulated (weak)	Tb927.9.9320	Full Length
LmxM.33.0060	Hypothetical protein	Cytoplasm (points)	Tb927.10.2480	Complex
LmxM.24.0700	Hypothetical protein	Cytoplasm posterior (points)	Tb927.11.5370	FAZ-ER
LmxM.14.1200	Phosphatidylserine synthase	Cytoplasm, lysosome	Tb927.7.3760	FAZ-ER
LmxM.02.0140	Ankyrin repeats (3 copies)/Zinc finger, C3HC4 type (RING finger)	Cytoplasm	Tb927.2.2360	Distal only
LmxM.27.1040	Stumpy formation signalling pathway protein	Cytoplasm (points)	Tb927.11.1640	Complex
LmxM.31.0220	Hypothetical protein	Cytoplasm (points)	Tb927.10.14400	Complex
LmxM.16.0490	Amastin surface glycoprotein	Cytoplasm (points), lysosome	Tb927.4.3520	Complex
	**Cytoskeleton**			
LmxM.29.2580	Reticulon domain protein	Cytoskeleton (anterior), cytoplasm (points)	Tb927.6.3840	FAZ-ER
LmxM.18.1080	PKAC3	Cytoskeleton, cytoplasm	Tb927.10.13010	Complex
LmxM.36.1920	FAZ-tip-localizing protein required for cytokinesis	Cytoskeleton (anterior), cleavage furrow	Tb927.10.6360	Distal only
LmxM.23.0080	Posterior and Ventral Edge protein 1	Cytoskeleton (posterior)	Tb927.8.2030	Complex
LmxM.34.3720	Hypothetical protein	Cytoskeleton (posterior)	Tb927.9.11540	Complex
LmxM.36.6960	CIF4	Cytoskeleton (anterior), cleavage furrow	Tb927.10.8240	Distal only
LmxM.21.0700	Phosphoserine/threonine/tyrosine-binding protein	Cytoplasm, cytoskeleton (anterior)	Tb927.10.1620	Complex
	**Complex**			
LmxM.21.1220	Furrow protein 1	Cell tip (posterior), cytoplasm point	Tb927.10.870	Complex
LmxM.31.2610	TOEFAZ1	Cell tip (posterior), cleavage furrow, nucleus	Tb927.11.15800	Distal only

LmxM.13.1590	Spindle assembly abnormal 4	Cell tip (anterior), basal body, kinetoplast, cytoskeleton (anterior)	Tb927.11.3300	Complex
LmxM.19.0680	FAZ7	Cytoskeleton (anterior), basal body, pro-basal body	Tb927.10.15390	Distal only
LmxM.19.0140	Associated kinase of Tb14-3-3	Cytoplasm, lysosome, kinetoplast	Tb927.10.14770	Complex
**ER**				
LmxM.22.1010	UAA transporter family	ER	Tb927.7.3070	FAZ-ER
LmxM.09.1050	VAMP-associated protein	ER	Tb927.11.13230	FAZ-ER
LmxM.11.1320	Major facilitator superfamily	ER	Tb927.11.6060	FAZ-ER
LmxM.31.2680	Hypothetical protein	ER	Tb927.11.15870	FAZ-ER
**Endocytic/lysosome**				
LmxM.29.2670	Autophagy-related protein 27	Endocytic, lysosome	Tb927.6.3940	Complex
**Flagellum**				
LmxM.36.0830	cAMP binding protein	Short line in flagellum beyond cell exit point	Tb927.10.5240	Distal only

Supplementary Table References

An, T., Zhou, Q., Hu, H., Cormaty, H., Li, Z. (2020). FAZ27 cooperates with FLAM3 and ClpGM6 to maintain cell morphology in *Trypanosoma brucei* . *Journal of Cell Science* , **133**, 1-26. https://doi.org/10.1242/jcs.245258

Aslett, M., Aurrecoechea, C., Berriman, M., Brestelli, J., Brunk, B.P., Carrington, M., Depledge, D.P., Fischer, S., Gajria, B., Gao, X., Gardner, M.J., Gingle, A., Grant, G., Harb, O.S., Heiges, M., Hertz-Fowler, C., Houston, R., Innamorato, F., Iodice, J., Kissinger, J.C., Kraemer, E., Li, W., Logan, F.J., Miller, J.A., Mitra, S., Myler, P.J., Nayak, V., Pennington, C., Phan, I., Pinney, D.F., Ramasamy, G., Rogers, M.B., Roos, D.S., Ross, C., Sivam, D., Smith, D.F., Srinivasamoorthy, G., Stoeckert, C.J., Subramanian, S., Thibodeau, R., Tivey, A., Treatman, C., Velarde, G., Wang, H. (2010). TriTrypDB: a functional genomic resource for the Trypanosomatidae. *Nucleic Acids Research* , **38**, 457–462. https://doi.org/10.1093/nar/gkp851

Blum, M., Chang, H.-Y., Chuguransky, S., Grego, T., Kandasaamy, S., Mitchell, A., Nuka, G., Paysan-Lafosse, T., Qureshi, M., Raj, S., Richardson, L., Salazar, G.A., Williams, L., Bork, P., Bridge, A., Gough, J., Haft, D.H., Letunic, I., Marchler-Bauer, A., Mi, H., Natale, D.A., Necci, M., Orengo, C.A., Pandurangan, A.P., Rivoire, C., Sigrist, C.J.A., Sillitoe, I., Thanki, N., Thomas, P.D., Tosatto, S.C.E., Wu, C.H., Bateman, A., Finn, R.D. (2021). The InterPro protein families and domains database: 20 years on. *Nucleic Acids Research* , **49**, 344–354. https://doi.org/10.1093/nar/gkaa977

Fischer, S., Brunk, B.P., Chen, F., Gao, X., Harb, O.S., Iodice, J.B., Shanmugam, D., Roos, D.S., Stoeckert, C.J. (2011). Using OrthoMCL to assign proteins to OrthoMCL-DB groups or to cluster proteomes into new ortholog groups. *Current Protocols in Bioinformatics* , **35**, 1-19. https://doi.org/10.1002/0471250953.bi0612s35

Hayes, P., Varga, V., Olego-fernandez, S., Sunter, J., Ginger, M.L., Gull, K. (2014). Modulation of a cytoskeletal calpain-like protein induces major transitions in trypanosome morphology. *The Journal of Cell Biology* , **206**, 377–384. https://doi.org/10.1083/jcb.201312067

Hilton, N.A., Sladewski, T.E., Perry, J.A., Pataki, Z., Sinclair-Davis, A.N., Muniz, R.S., Tran, H.L., Wurster, J.I., Seo, J., De Graffenried, C.L. (2018). Identification of TOEFAZ1-interacting proteins reveals key regulators of *Trypanosoma brucei* cytokinesis. *Molecular Microbiology* , **109**, 306–326. https://doi.org/10.1111/mmi.13986

Hu, H., Zhou, Q., Li, Z. (2015a). SAS-4 in *Trypanosoma brucei* controls life cycle transitions by modulating the length of the flagellum attachment zone filament. *Journal of Biological Chemistry* , **290**, 30453–30463. https://doi.org/10.1074/jbc.M115.694109

Hu, H., Zhou, Q., Li, Z. (2015b). A novel basal body protein that is a polo-like kinase substrate is required for basal body segregation and flagellum adhesion in *Trypanosoma brucei* . *Journal of Biological Chemistry* , **290**, 25012–25022. https://doi.org/10.1074/jbc.M115.674796

Kurasawa, Y., Hu, H., Zhou, Q., Li, Z. (2018). The trypanosome-specific protein CIF3 cooperates with the CIF1 protein to promote cytokinesis in *Trypanosoma brucei* . *Journal of Biological Chemistry* , **293**, 10275–10286. https://doi.org/10.1074/jbc.RA118.003113

Lacomble, S., Vaughan, S., Deghelt, M., Moreira-leite, F.F., Gull, K. (2012). A *Trypanosoma brucei* protein required for maintenance of the flagellum attachment zone and flagellar pocket ER domains. *Protist* , **163**, 602–615. https://doi.org/10.1016/j.protis.2011.10.010

Li, Z., Lee, J.H., Chu, F., Burlingame, A.L., Günzl, A., Wang, C.C. (2008). Identification of a novel chromosomal passenger complex and its unique localization during cytokinesis in *Trypanosoma brucei* . *PLoS ONE* , **3**, 1-13. https://doi.org/10.1371/journal.pone.0002354

Li, Z., Wang, C.C. (2008). KMP-11, a basal body and flagellar protein, is required for cell division in *Trypanosoma brucei* . *Eukaryotic Cell* , **7**, 1941–1950. https://doi.org/10.1128/EC.00249-08

McAllaster, M.R., Ikeda, K.N., Lozano-Nunez, A., Anrather, D., Unterwurzacher, V., Gossenreiter, T., Perry, J.A., Crichley, R., Mercadante, C.J., Vaughan, S., de Graffenried, C.L. (2015). Proteomic identification of novel cytoskeletal proteins associated with TbPLK, an essential regulator of cell morphogenesis in *Trypanosoma brucei* . *Molecular Biology of the Cell* , **26**, 1–56. https://www.molbiolcell.org/doi/10.1091/mbc.E15-04-0219

Morriswood, B., Havlicek, K., Demmel, L., Yavuz, S., Sealey-Cardona, M., Vidilaseris, K., Anrather, D., Kostan, J., Djinović-Carugo, K., Roux, K.J., Warren, G. (2013). Novel bilobe components in *Trypanosoma brucei* identified using proximity-dependent biotinylation. *Eukaryotic Cell* , **12**, 356-367. https://doi.org/10.1128/EC.00326-12

Oberholzer, M., Langousis, G., Nguyen, H.T., Saada, E.A., Shimogawa, M.M., Jonsson, Z.O., Nguyen, S.M., Wohlschlegel, J.A., Hill, K.L. (2011). Independent analysis of the flagellum surface and matrix proteomes provides insight into flagellum signaling in mammalian-infectious *Trypanosoma brucei* . *Molecular and Cellular Proteomics* , **10**, 1–14. https://doi.org/10.1074/mcp.M111.010538

Rotureau, B., Blisnick, T., Subota, I., Julkowska, D., Cayet, N., Perrot, S., Bastin, P. (2014). Flagellar adhesion in *Trypanosoma brucei* relies on interactions between different skeletal structures in the flagellum and cell body. *Journal of Cell Science* , **127**, 204–215. https://doi.org/10.1242/jcs.136424

Sun, S.Y., Wang, C., Yuan, Y.A., He, C.Y., 2013. An intracellular membrane junction consisting of flagellum adhesion glycoproteins links flagellum biogenesis to cell morphogenesis in *Trypanosoma brucei* . *Journal of Cell Science* , **126**, 520–531. https://doi.org/10.1242/jcs.113621

Sunter, J.D., Varga, V., Dean, S., Gull, K. (2015). A dynamic coordination of flagellum and cytoplasmic cytoskeleton assembly specifies cell morphogenesis in trypanosomes. *Journal of Cell Science* , **128**, 1580–1594. https://doi.org/10.1242/jcs.166447

Vaughan, S., Kohl, L., Ngai, I., Wheeler, R.J., Gull, K. (2008). A repetitive protein essential for the flagellum attachment zone filament structure and function in *Trypanosoma brucei* . *Protist* , **159**, 127–136. https://doi.org/10.1016/j.protis.2007.08.005

Zhou, Q., An, T., Pham, K.T.M., Hu, H., Li, Z. (2018a). The CIF1 protein is a master orchestrator of trypanosome cytokinesis that recruits several cytokinesis regulators to the cytokinesis initiation site. *Journal of Biological Chemistry* , **293**, 16177–16192. https://doi.org/10.1074/jbc.RA118.004888

Zhou, Q., Gu, J., Lun, Z.-R., Ayala, F.J., Li, Z. (2016). Two distinct cytokinesis pathways drive trypanosome cell division initiation from opposite cell ends. *PNAS* , **113**, 3287–3292. https://doi.org/10.1073/pnas.1601596113

Zhou, Q., Hu, H., He, C.Y., Li, Z. (2015). Assembly and maintenance of the flagellum attachment zone filament in *Trypanosoma brucei* . *Journal of Cell Science* , **128**, 2361–2372. https://doi.org/10.1242/jcs.168377

Zhou, Q., Hu, H., Li, Z. (2016). An EF-hand-containing protein in Trypanosoma brucei regulates cytokinesis initiation by maintaining the stability of the Cytokinesis Initiation Factor CIF1. *Journal of Biological Chemistry* , **291**, 14395–14409. https://doi.org/10.1074/jbc.M116.726133

Zhou, Q., Lee, K.J., Kurasawa, Y., Hu, H., An, T., Li, Z. (2018b). Faithful chromosome segregation in *Trypanosoma brucei* requires a cohort of divergent spindle-associated proteins with distinct functions. *Nucleic Acids Research* , **46**, 8216–8231. https://doi.org/10.1093/nar/gky557

Zhou, Q., Liu, B., Sun, Y., He, C.Y. (2011). A coiled-coil- and C2-domain-containing protein is required for FAZ assembly and cell morphology in *Trypanosoma brucei* . *Journal of Cell Science* , **124**, 3848–3858. https://doi.org/10.1242/jcs.087676
